# Pyrazolyl amide-chalcones conjugates: Synthesis and antikinetoplastid activity

**DOI:** 10.1007/s00210-024-03524-7

**Published:** 2024-10-21

**Authors:** Devesh S. Agarwal, Richard M. Beteck, Dorien Mabille, Guy Caljon, Lesetja J. Legoabe

**Affiliations:** 1 Centre of Excellence for Pharmaceutical Sciences, North-West University, Potchefstroom, 2520 South Africa; 2 Laboratory of Microbiology, Parasitology and Hygiene, Infla-Med Centre of Excellence, University of Antwerp, Antwerp, Belgium

**Keywords:** Pyrazoles, Chalcones, Amides, Antikinetoplastid, Drug likeliness properties

## Abstract

A series of novel pyrazolyl amide-chalcones conjugates was synthesized in five steps and evaluated against a range of medically important kinetoplastid parasites including *Trypanosoma cruzi*, *Trypanosoma brucei brucei*, *Trypanosoma brucei rhodesiense* and *Leishmania infantum*. In addition, the series was also tested for in vitro cytotoxicity activity against human lung fibroblasts and primary mouse macrophages. Among all synthetised compounds, **9b** was found to be the most active against *T. b. brucei* with an IC_50_ value of 0.51 ± 0.06 μM. Against *T. b. rhodesiense*, **9n** was found to be the most potent with an IC_50_ value of 0.46 ± 0.07 μM. While against *L. infantum*, **9a** was found to be most active with an IC_50_ value of 7.16 ± 1.88 μM. Based on the results and SAR, further modifications will be carried out to increase potency.

**Graphical Abstract:**

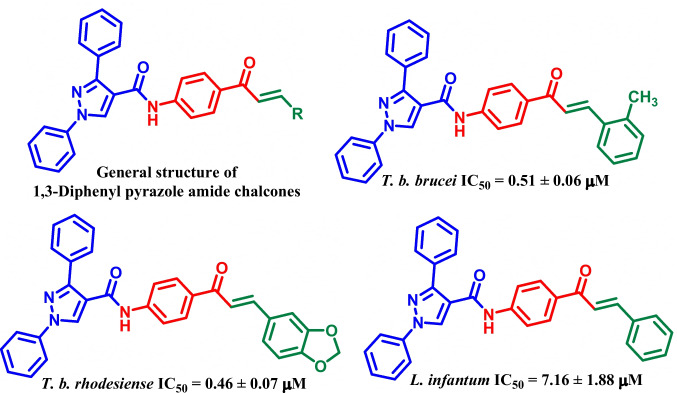

**Supplementary Information:**

The online version contains supplementary material available at 10.1007/s00210-024-03524-7.

## Introduction

Pyrazoles constitutes a versatile class of five-membered heterocyclic compounds of the azole family (Fustero et al. 2011). Pyrazole-containing molecules have attracted interest from various scientists across the globe for their diverse pharmacological properties such as anticancer, (Zhang et al. 2023) antifungal, (Bennani 2022) anti-inflammatory, (Kaur et al. 2022) antiviral, (Karati et al. 2022) anti-tuberculosis, (Xu et al. 2017) antidepressant, (Ansari et al. 2017) antioxidant, (Silva et al. 2018) anti-HIV activity (Kumar et al. 2022) and as inhibitors of protein glycation (Ansari et al. 2017; Ebenezer et al. 2022).

Chalcones, another versatile compound class of both natural and synthetic origin, have been found to possess a range of beneficial pharmacological properties such as antibacterial, anticancer, anthelmintic, antiulcer, immunosuppressive, antiprotozoal, cytotoxic, amoebicidal, antiviral and insecticidal activities (Rammohan et al. 2020; Salehi et al. 2021; Elkanzi et al. 2022).

Neglected tropical diseases (NTDs), which includes Chagas disease (CD) or American trypanosomiasis (caused by *T. cruzi*), sleeping sickness or human African trypanosomiasis (HAT; caused by *T. b. gambiense* in West and Central Africa and *T. b. rhodesiense* in East Africa) (Simarro et al. 2012) and leishmaniasis (caused by several *Leishmania* species) are huge public health burdens in several parts of the world (Ackley et al. 2021; Ghorai 2023; WHO 2023a). In the case of leishmaniasis, WHO declared that 350 million people in 88 countries are at risk of contracting leishmaniasis and 0.6 to 1.0 million cases of cutaneous leishmaniasis (CL) are recorded every year (Alvar et al. 2012; WHO 2023b). In case of CD, six million people are affected worldwide (de Sousa et al. 2024). The current treatments for these diseases are not very effective, partly attributed to the fact that most of the available drugs are difficult to administer and have severe side effects (Murray 2001). For instance, nifurtimox (Nfx) and benznidazole (Bnz), the two drugs available for CD, have relatively low efficacy, severe adverse effects and are ineffective against drug resistant *T. cruzi* strains (Brun et al. 2010; Bernardes et al. 2013). In the case of leishmaniasis, first-line treatment in most areas still relies on heavy metals such as antimony which exhibit substantial toxicity. The second-line drugs such as amphotericin B, pentamidine, paromomycin and miltefosine also have numerous side effects. Moreover, there emergence and spread of *Leishmania* strains that are resistant to these drugs is another major public health concern (Peixoto et al. 2011).

**Fig. 1:**
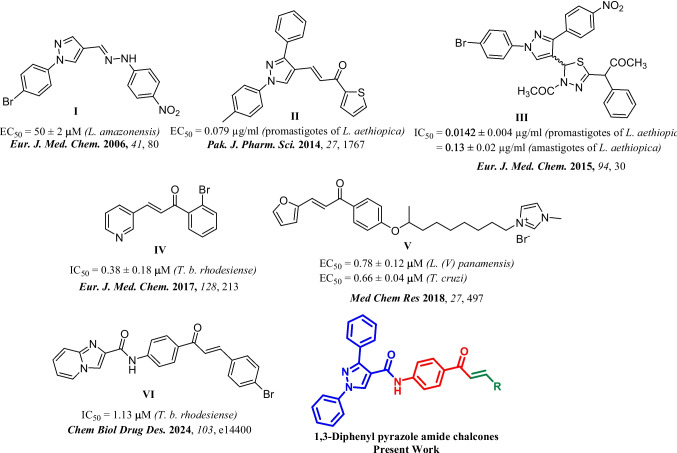
Representative examples and rationale design for the synthesis of pyrazolyl amide-chalcones conjugates

To overcome current challenges associated with the treatment of these diseases, extensive research has been done for the development of novel and efficient agents against the aforementioned diseases. For example, the Amaral group reported synthesis of 1*H*-pyrazole-4-carbohydrazides and studied their leishmanicidal activity. The most active compound (**I**, Fig. 1) was found to possess an IC_50_ value of 50 µM against *L. amazonensis* (Bernardino et al. 2006). In 2014, Bekhit et al. reported synthesis of 1*H*-pyrazole derivatives as antimalarial and antileishmanial agents. The most potent derivative (**II**, Fig. 1) exhibited an EC_50_ value of 0.079 µg/mL against promastigotes of *L. aethiopica*.(Bekhit et al. 2014) In 2015, the same group reported synthesis of pyrazoles hybridized thiazoles, thiazolidinones, 1,3,4-thiadiazoles and pyrazolines as antimalarial and antileishmanial agents. The most active derivative (**III**, Fig. 1) was found to exhibit an IC_50_ value of 0.0142 µg/mL and 0.13 µg/ mL against promastigotes and amastigotes of *L. aethiopica*, respectively (Bekhit et al. 2015). Bhambra group reported synthesis of pyridylchalcones as antitrypanosomal agents against *T. b. rhodesiense*. The most active analogue (**IV**, Fig. 1) was found to possess an IC_50_ value of 0.38 μM against *T. b. rhodesiense* (Bhambra et al. 2017). In 2016, Wilson I. Cardona group reported synthesis of furanchalcone–quinoline, furanchalcone–chromone and furanchalcone–imidazole hybrids. Among all the chalcone derivatives, **V** was found to be most potent with an EC_50_ value of 0.78 µM and 0.66 µM against *L. panamensis* and *T. cruzi*, respectively (García et al. 2018).

Recently, our group reported synthesis of imidazo[1,2-*a*]pyridine-chalcone amides and studied their antikinetoplastid activity. The most active compound (**VI**, Fig. 1) was found to possess an IC_50_ value of 1.13 µM against *T. b. rhodesiense* (Agarwal et al. 2024).

**Fig. 2:**
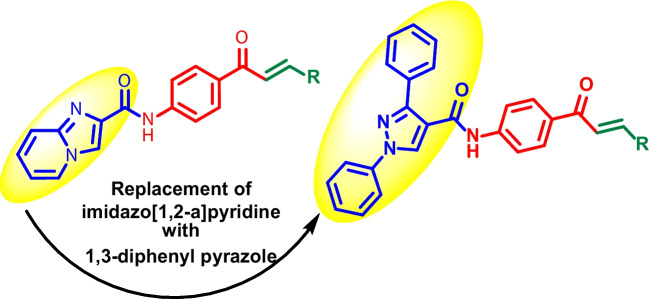
Rationale for the synthesis of pyrazolyl amide-chalcones conjugates (**9a**-**n**)

Based on the above examples and our previous report (Agarwal et al. 2024), we attempted to replace imidazo[1,2-*a*]pyridine moiety with a 1,3-diphenyl pyrazole moiety (Fig. 2). The diversified application of pyrazole moiety in medicinal chemistry (Karrouchi et al. 2018) prompted us to substitute imidazo[1,2-*a*]pyridine moiety from our previous work with pyrazole and study their activity against *T. cruzi*, *T. b. brucei*, *T. b. rhodesiense* and *L. infantum*.

**Scheme 1:**
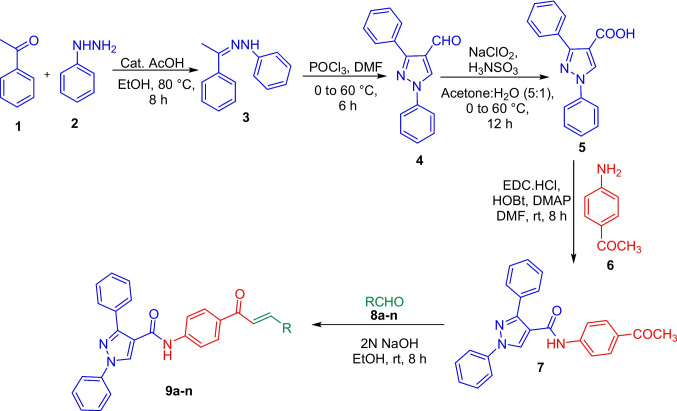
Synthesis of pyrazolyl amide-chalcones conjugates **9a-n**

In our continuous efforts towards the development of novel antikinetoplastid agents, (Agarwal et al. 2024) we herein report the synthesis of pyrazolyl amide-chalcones conjugates (**9a**-**n**) Scheme 1. The synthesized analogues were tested for their antikinetoplastid activity against *T. cruzi*, *T. b. brucei*, *T. b. rhodesiense* and *L. infantum*. In addition, the synthesized analogues were also tested for their in vitro cytotoxicity against human lung fibroblasts (MRC-5) and primary mouse macrophages (PMMs).

## Result and discussion

### Chemistry

In the present study, novel pyrazolyl amide-chalcones conjugates were synthesized via a series of steps (**9a-n**, Scheme 1). The synthesis commenced with the reaction of acetophenone (**1**) with phenyl hydrazine (**2**) in EtOH at 80 °C to give 1-phenyl-2-(1-phenylethylidene)hydrazine (**3**) in 70% yield (Reddy et al. 2015). Cyclization and formylation of 1-phenyl-2-(1-phenylethylidene)hydrazine (**3**) using POCl_3_ in DMF gave 1,3-diphenyl-1*H*-pyrazole-4-carbaldehyde (**4**) in 76% yield (Prakash et al. 2006). The oxidation of **4** was carried out using sodium chlorite (NaClO_2_) and sulfamic acid (NH_3_SO_3_) in acetone: water (5:1) at 60 °C to give 1,3-diphenyl-1*H*-pyrazole-4-carboxylic acid (**5**) as off-white solid. Later, **5** was coupled with 1-(4-aminophenyl)ethan-1-one (**6**) using EDC.HCl/HOBt in DMF at room temperature (rt) to give *N*-(4-acetylphenyl)-1,3-diphenyl-1*H*-pyrazole-4-carboxamide (**7**) in 79% yield (Scheme 1) (Agarwal et al. 2016). The corresponding ^1^H NMR showed the presence of amide NH proton at *δ* 10.52 and methyl group (-COCH_3_) proton at *δ* 2.55 and the corresponding ^13^C NMR showed carbonyl group (C = O) of amide and ketone at *δ* 161.79 and 196.50, respectively confirmed the formation of **7**.

Finally, **7** was reacted with different aldehydes (**8**) in EtOH using 2N NaOH to give corresponding pyrazolyl amide-chalcones conjugates (**9a-n**) in excellent yields (Scheme 1). The structures of **4**, **5**, **7** and **9a**-**n** were characterized using ^1^H NMR, ^13^C NMR and HRMS.

### Pharmacology

All the synthesized compounds **4**, **5**, **7** & **9a-n** were evaluated for antikinetoplastid activity against intracellular *T. cruzi*, the blood stream trypomastigotes of *T. b. brucei*, and *T. b. rhodesiense,* and against *L. infantum* (intracellular amastigotes). The synthesized analogues were also evaluated for their cytotoxicity against human lung fibroblasts (MRC-5) and primary mouse macrophages (PMMs). Compounds, **4**, **5** and **7** which lacked chalcone moiety were found to be inactive. This observation emphasised that the chalcone moiety is essential for the antikinetoplastid activity of this compounds set.

With respect to growth inhibitory activity against *T. cruzi*, **9f** (2-Cl) was the only active and nontoxic compound, with an IC_50_ value of 13.43 μM against *T. cruzi*, and IC_50_ value of > 64 μM against both human lung fibroblasts (MRC-5) and primary mouse macrophages (PMMs).

**Fig. 3:**
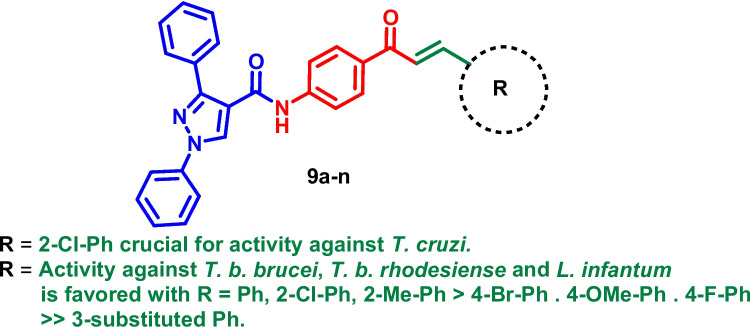
SAR of pyrazolyl amide-chalcones conjugates (**9a**-**n**)

This series of compounds demonstrates activity against *T. b. brucei* that ranges from an IC_50_ value of 0.51 to > 64 μM. In general, the compounds displayed similar activities against *T. brucei* and *T. b. rhodesiense* except for compound **9d**, **9 h**, **9 l**, and **9n** which were approximately 4- to 7-times more potent against *T. b. rhodesiense*. The effect of the substituted aryl group (R) on the anti-*T. brucei* activity was as follows: compounds with a phenyl ring substituted in the 2-position [2-Me (**9b**, IC_50_ = 0.51 μM) > 2-Cl (**9f**, IC_50_ = 0.60 μM)] were more active than 4-position [4-Br (**9e**, IC_50_ = 1.13 μM)≈ 4-OMe (**9d**, IC_50_ = 1.64 μM) ≈ 4-F (**9i**, IC_50_ = 0.78 μM)] >  > 3-position [3-OMe (**9d**, IC_50_ = 1.64 μM) > 3-F (**9 h**, IC_50_ = 6.36 μM). This data is summarized in Table 1 and Fig. 3.

**Table 1:**
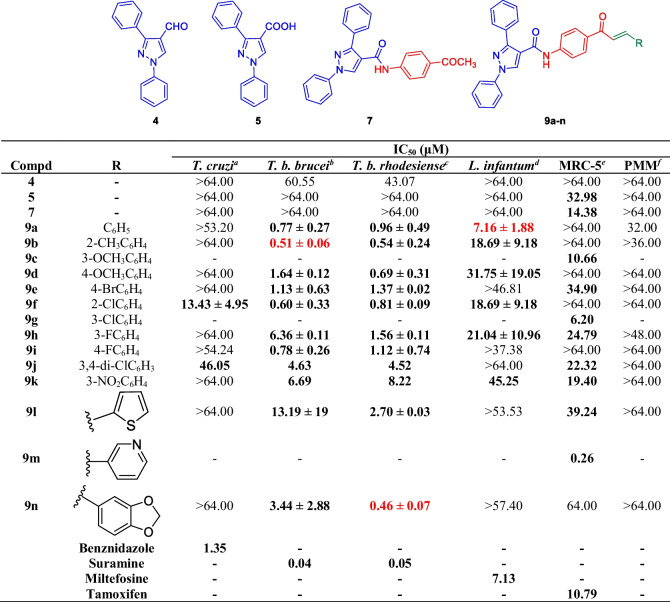
Antikinetoplastid and cytotoxic activities (IC_50_, μM) of synthesized compounds (**4**, **5**, **7** & **9a-n**)

^*a*^*Trypanosoma cruzi* (intracellular amastigotes)*, *^*b*^*Trypanosoma brucei brucei *(blood stream trypomastigotes)*, *^*c*^*Trypanosoma brucei rhodesiense *(blood stream trypomastigotes)*, *^*d*^* Leishmania infantum (*intracellular amastigotes*), *^*e*^lung fibroblast, ^*f*^primary mouse microphages, ^*g*^- Not tested, Bold values in the table represents derivatives with moderate to good activity while the values marked in red bold are the most potent derivatives against a particular pathogen.

With respect to the activity against *T. b. rhodesiense*, derivatives bearing electron-donating group **9b** (2-Me, IC_50_ = 0.54 μM) ≈**9d** (4-OMe, IC_50_ = 0.69 μM) and **9a** (Ph, IC_50_ = 0.96 μM) were found to be active. Among the halogen substituted derivatives, **9e** (4-Br, IC_50_ = 1.37 μM), **9f** (2-Cl, IC_50_ = 0.81 μM), **9 h** (3-F, IC_50_ = 1.56 μM), **9i** (4-F, IC_50_ = 1.12 μM) and **9j** (3,4-di-Cl, IC_50_ = 4.52 μM) were found to be active. With respect to the heterocyclic derivatives, **9n** ((*4-(3-(benzo[d][1,3]dioxol-5-yl)*, IC_50_ = 0.46 μM was found to be the most active. While, **9 l** was found to exhibit an IC_50_ value of 2.70 μM.

Against *L. infantum*, **9a** was found to be the most active with an IC_50_ value of 7.16 μM. The antileishmanial activity of **9a** is comparable to that of the reference drug, miltefosine (IC_50_ = 7.13 μM). Compounds **9b**, **9d**, **9f** and **9 h** also demonstrated activity, although moderate to weak, with IC_50_ values of 18.69, 31.75, 18.69 and 21.04 μM, respectively.

Compounds **9c**, **9 g** and **9 m** were found to be cytotoxic in nature with CC_50_ values of 10.66, 6.20 and 0.26 μM against MRC-5, respectively. These compounds were not tested for their antikinetoplastid activity. With respect to the cytotoxicity against PMM, all analogues were found to exhibit non-cytotoxic properties with CC_50_ values of more than 32 μM.

As per literature report, it can be postulated that potent activity of the chalcone derivatives can be attributed to the delocalization of electron density in the (C = C–C = O) system. In addition, the *α*, *β* unsaturated carbonyl functional group in chalcone acts as potential Michael acceptor, allowing them to interact with sulfhydryl of cysteine residue or other thiol groups. This interaction is believed to be important for their biological activities (Thapa et al. 2021).

Chalcones have two electrophilic reactive centers, allowing them to act as synthons for further derivatization, e.g. an attack on the carbonyl group (1,2-addition) or engaging the *β*-carbon (1,4-conjugate addition) (Tukur et al. 2022; Mitrev et al. 2016).

**Fig. 4:**
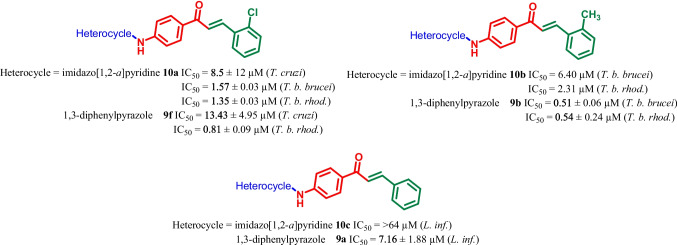
Comparison of few imidazo[1,2-*a*]pyridine-chalcone conjugates (10a-c) and pyrazolyl amide-chalcones conjugates (**9a**-**n**) as antikinetoplastid agents

It can be concluded that replacement of imidazo[1,2-a]pyridine with a 1,3-diphenylpyrazole heterocycle had varied effect on the antikinetoplastid activity of the chalcone derivatives. For instance, with respect to the activity against *T. cruzi*, *T. b. brucei* and *T. b. rhodesiense*, the replacement of imidazo[1,2-a]pyridine with a 1,3-diphenylpyrazole had a very minor effect on the activity (Fig. 4). For instance, **10a** (IC_50_ = 8.5 μM) was found to be more active as compared to its counterpart **9f** (IC_50_ = 13.43 μM) against *T. cruzi*. While against *T. b. rhodesiense*, both **10a** (IC_50_ = 1.35 μM), **10b** (IC_50_ = 2.31 μM), **9f** (IC_50_ = 0.81 μM) and **9b** (IC_50_ = 0.54 μM) were found to have similar activity.

With respect to the activity against *T. brucei*, replacement of imidazo[1,2-a]pyridine with a 1,3-diphenylpyrazole heterocycle let to enhanced antikinetoplastid activity. This is demonstrated when comparing both imidazo[1,2-a]pyridine chalcone derivatives **10a** (IC_50_ = 1.57 ± 0.03 μM) and **10b** (IC_50_ = 6.40 μM) against 1,3-diphenylpyrazole chalcone derivative **9b** (IC_50_ = 0.51 ± 0.06 μM).

In contrast, 1,3-diphenylpyrazole chalcone derivatives were active in the micromolar range against intracellular amastigotes of *L. infantum* whereas the imidazo[1,2-a]pyridine counterparts were totally inactive against this parasite. For example, **9a** (IC_50_ = 7.16 ± 1.88 μM) was found to more active as compared to the imidazo[1,2-a]pyridine chalcone derivative **10c** (IC_50_ =  > 64.00 μM) (Fig. 4) (Agarwal et al. 2024).

### Drug likeliness properties and drug score predictions

Drug likeliness properties of the synthesized compounds **4**, **5**, **7** and **9a-n** were calculated to predict the absorption, distribution, metabolism and excretion (ADME) properties online using Molinspiration cheminformatics software (Table 2) (Agarwal et al. 2018). In addition, the drug likeness score and percentage absorption were also calculated using MolSoft software online and using the formula %ABS = 109 − (0.345 × TPSA), respectively (Zhao et al. 2002). Among all the derivatives, the most active derivatives **9b**, **9n** and **9a** against *T. b. brucei*, *T. b. rhodesiense* and *L. infantum* were found to possess TPSA less than 160 Å^2^ but > 40 Å^2^ making them excellent candidates for intestinal absorption (Table 2). With respect to the drug likeliness score, **9a** and **9b** were found to have a positive drug likeness score of 0.18 and 0.02, whereas **9n** was found to have a negative score of -0.11 (Table 2). The positive value and the value closer to 1 of drug likeness score suggest the molecule to be more aligned to behave like a drug. For instance, **9a** and **9b** could be ideal drug candidates as they have a positive drug likeness score while, **9n** has a negative drug likeness score although the derivative is found to be more potent in vitro (Lalitha and Sivakamasundari 2010).

**Table 2:** Drug likeliness properties, % ABS and drug score predictions of synthesized compounds (**4**, **5**, **7** & **9a-n**)

Compd	miLogP^*a*^	TPSA^*b*^	natoms^*c*^	MW^*d*^	nHBA^*e*^ (O & N)	nHBD^*f*^ (OH & NH)	nviolations^*g*^	nrotb^*h*^	volume^*i*^	% ABS^*j*^	Drug-likeness Score^*k*^
4	3.05	34.90	19	248.28	3	0	0	3	227.06	96.95	-1.38
5	2.83	55.12	20	264.28	4	1	0	3	235.07	89.98	-0.76
7	4.05	63.99	29	381.44	5	1	0	5	346.41	86.92	0.00
9a	**6.03**	**63.99**	**36**	**469.54**	**5**	**1**	**1**	**7**	**428.68**	**86.92**	**0.18**
9b	**6.25**	**63.99**	**37**	**483.57**	**5**	**1**	**1**	**7**	**445.24**	**86.92**	**0.02**
9c	6.06	73.23	38	499.57	6	1	1	8	454.22	83.73	0.50
9d	6.08	73.23	38	499.57	6	1	1	8	454.22	83.73	0.31
9e	6.83	63.99	37	548.44	5	1	2	7	446.56	86.92	0.23
9f	6.48	63.99	37	503.99	5	1	2	7	442.21	86.92	0.17
9 g	6.68	63.99	37	503.99	5	1	2	7	442.21	86.92	0.48
9 h	6.17	63.99	37	487.53	5	1	1	7	433.61	86.92	0.40
9i	6.19	63.99	37	487.53	5	1	1	7	433.61	86.92	0.40
9j	7.31	63.99	38	538.43	5	1	2	7	455.75	86.92	0.43
9 k	5.96	109.82	39	514.54	8	1	2	8	452.01	71.11	0.16
9 l	5.75	63.99	35	475.57	5	1	1	7	419.39	86.92	0.27
9 m	4.61	76.89	36	470.53	6	1	0	7	424.52	82.47	0.64
9n	**5.92**	**82.46**	**39**	**513.55**	**7**	**1**	**2**	**7**	**452.60**	**80.55**	**-0.11**

^*a*^ Logarithm of compound partition coefficient between *n*-octanol and water, ^*b*^ Topological polar surface area (Å^2^), ^*c*^ Number of atoms in the molecule, ^*d*^ Molecular weight of the molecules. ^*e*^ Number of hydrogen bond acceptors, ^*f*^ Number of hydrogen bond donors, ^*g*^Number of violations of Lipinski’s rule, ^*h*^ Number of rotatable bonds, ^*i*^ Volume of the molecule, ^*j*^Percentage absorption calculated using the formula %ABS = 109 − (0.345 × TPSA), ^*k*^Drug-likeness Score calculated using molsoft. Bold values represent the molecular properties of the most active derivatives against particular pathogens

## Conclusions

In summary, we have designed and synthesized a series of pyrazolyl amide-chalcones conjugates in good to excellent yield. All the compounds were characterized using ^1^H NMR, ^13^C NMR and HRMS. The compounds were tested for antiparasitic activity against *T. cruzi*, *T. b. brucei*, *T. b. rhodesiense* and *L. infantum*. For their in vitro cytotoxicity, they were tested against human lung fibroblasts (MRC-5) and primary mouse macrophages (PMMs). Among all the compounds, **9b** was found to be the most active against *T. b. brucei* with an IC_50_ value of 0.51 μM. Against *T. b. rhodesiense* **9n** was found to be most active with an IC_50_ value of 0.46 μM. Against *L. infantum*, **9a** was found to be most active with an IC_50_ value of 7.16 μM. These active compounds (**9b**, **9n** and **9a)** were found to be non-cytotoxic against human lung fibroblasts (MRC-5) and primary mouse macrophages (PMMs). Thus, the present study revealed that pyrazolyl amide-chalcones conjugates could serve as candidates for hit-to-lead optimization and selection of molecules for in vivo studies.

## Materials and methods

### General

All the chemicals were purchased from various chemical suppliers including Sigma-Aldrich, Ace, Rochelle, Ambeed and used without further purification. The progress of the reactions was monitored using thin layer chromatography (TLC) on Merck 60F_254_ silica gel plates supported on 0.20 mm thick aluminium sheets. Nuclear magnetic resonance (NMR) spectra were recorded on a Bruker Avance III 600 spectrophotometer at 600 MHz (for ^1^H) and 150 MHz (for ^13^C). The ^1^H NMR chemical shifts (*δ*) were reported in parts per million (ppm) and were measured relative to residual deuterochloroform (CDCl_3_) (7.26 ppm) or DMSO-*d*_*6*_ (2.5 ppm). The ^13^C NMR chemical shifts (*δ*) were reported in ppm relative to deuterochloroform (CDCl_3_) (77.0 ppm) or DMSO-*d*_*6*_ (39.5 ppm). All coupling constants *J* were reported in Hz. The following abbreviations were used to describe peak splitting patterns: s = singlet, d = doublet, t = triplet, dd = doublet of doublet, m = multiplet and brs = broad singlet. High resolution mass spectra were recorded on an Agilent Technologies micrOTOF-Q II 2010390 by using atmospheric pressure chemical ionization (APCI) in positive ion mode. Melting points were obtained using Buchi B-545 melting point apparatus and are uncorrected. 1-phenyl-2-(1-phenylethylidene)hydrazine (**3**) was synthesized according to the literature reports (Reddy et al. 2015).

### Chemistry

#### General procedure for the synthesis of 1,3-diphenyl-1H-pyrazole-4-carbaldehyde (4)

To a stirred solution of DMF (60 mL), POCl_3_ (10.93 g, 0.07 mol) was added dropwise at 0 °C and the mixture was stirred at 0 °C for 0.5 h. Subsequently, **3** (10 g, 0.05 mol) was added portion wise at 0 °C, after which the reaction was slowly brought to rt (room temperature) and finally heated at 60 °C for 8 h. After the completion of the reaction, the reaction mixture was cooled and quenched by adding crushed ice and saturated solution of NaHCO_3_ which resulted in the formation of the precipitate. The resulted precipitate was filtered under suction to give the crude product, which was recrystallized using EtOH to give pure product **4**.

Off-white solid, 8.9 g (76% yield), m.p.: 140–142 °C (Lit. m.p.: 137–138 °C) (Prakash et al. 2006), ^1^H NMR (600 MHz, DMSO-*d*_*6*_) *δ* 10.00 (s, 1H), 9.32 (s, 1H), 8.00 (d, *J* = 7.9 Hz, 2H), 7.93 (d, *J* = 7.2 Hz, 2H), 7.58 (t, *J* = 7.6 Hz, 2H), 7.51 (dd, *J* = 16.4, 9.2 Hz, 3H), 7.44 (t, *J* = 7.3 Hz, 1H).^13^C NMR (150 MHz, DMSO-*d*_*6*_) *δ* 184.5, 152.6, 138.5, 134.8, 131.2, 129.6, 129.1, 128.6, 128.5, 127.6, 112.1, 119.2. HRMS (APCI): m/z calcd for C_16_H_13_N_2_O^+^ 249.1022 [M + H]^+^, found 249.1031.

#### General procedure for the synthesis of 1,3-diphenyl-1H-pyrazole-4-carboxylic acid (5)

To a stirred solution of 1,3-diphenyl-1*H*-pyrazole-4-carbaldehyde (**4**, 5 g, 0.020 mol) in acetone:H_2_O (5:1) at 0 °C, sodium chlorite (2.00 g,, 0.022 mol) and sulfamic acid (2.14 g, 0.022 mol) were added and the reaction was stirred at 60 °C for 12 h. After the completion of the reaction, the reaction mixture was cooled, and acetone was removed. The resulted reaction mixture was extracted using ethyl acetate (200 × 2). The combined ethyl acetate layer was concentrated under reduced pressure to give crude product, which was purified by recrystallization using EtOH to give pure 1,3-diphenyl-1*H*-pyrazole-4-carboxylic acid (**5**).

Off-white solid, 4.62 g (87% yield), m.p.: 200–201 °C (Lit. m.p.: 201.8–203.8 °C) (Zou et al. 2023)°C (Lit. m.p.: 137–138 °C), ^1^H NMR (600 MHz, DMSO-*d*_*6*_) *δ* 9.06 (s, 1H), 7.97 (d, *J* = 8.0 Hz, 2H), 7.83 (d, *J* = 6.8 Hz, 2H), 7.54 (t, *J* = 7.9 Hz, 2H), 7.48 – 7.36 (m, 4H). ^13^C NMR (150 MHz, DMSO-*d*_*6*_) 163.8, 152.9, 138.8, 133.6, 132.2, 129.6, 129.2, 128.5, 127.8, 127.3, 119.1, 114.0. HRMS (APCI): m/z calcd for C_16_H_13_N_2_O_2_^+^ 265.0972 [M + H]^+^, found 265.0963 [M + H]^+^.

#### General procedure for the synthesis of N-(4-acetylphenyl)-1,3-diphenyl-1H-pyrazole-4-carboxamide (7)

To a stirred solution of 1,3-diphenyl-1*H*-pyrazole-4-carboxylic acid (**5**, 5 g, 0.018 mol) in DMF (10 mL), DMAP (2.54 g, 0.020 mol) was added at rt and subsequently, EDC.HCl (5.3 g, 0.028 mol) and HOBt (2.89 g, 0.0189 mol) was added. The reaction was stirred at rt for 0.5 h, after that 1-(4-aminophenyl)ethan-1-one (**6**, 2.55 g, 0.0189 mol) was added and the reaction was stirred at rt for 8 h. After the completion of the reaction, the reaction was quenched by adding water and the aqueous layer was extracted using ethyl acetate (200 mL) twice. The combined organic layer was dried over anhydrous Na_2_SO_4_ and concentrated under reduced pressure to give crude product. The crude product was purified by recrystallized using EtOH to give pure **7**.

Off-white solid, 5.70 g (79% yield), m.p.: 149–150 °C, ^1^H NMR (600 MHz, DMSO-*d*_*6*_) *δ* 10.52 (s, 1H), 9.15 (s, 1H), 8.00 – 7.87 (m, 4H), 7.84 (d, *J* = 8.1 Hz, 4H), 7.59 (t, *J* = 7.7 Hz, 2H), 7.48 – 7.39 (m, 4H), 2.55 (s, 3H).^13^C NMR (150 MHz, DMSO-*d*_*6*_) 196.5, 161.8, 151.3, 130.9, 129.7, 129.4, 128.4, 128.2, 128.1, 127.2, 118.9, 118.7, 117.5, 26.4. HRMS (APCI): m/z calcd for C_24_H_20_N_3_O_2_^+^ 382.1550 [M + H]^+^, found 382.1552.

#### General procedure for the synthesis of pyrazolyl amide-chalcones conjugates (9a-n)

To a stirred solution of *N*-(4-acetylphenyl)-1,3-diphenyl-1*H*-pyrazole-4-carboxamide (**7**, 150 mg, 0.393 mmol) in ethanol (5 mL), 2N NaOH (2 mL) was added at rt and the reaction was stirred for 10 min. Subsequently, different aldehydes were added, and the reaction was stirred at rt for 8 h. After completion of the reaction, the reaction mixture was quenched by adding water and the resulted precipitate is filtered under suction. The resulted precipitate is consequently washed with water and finally recrystallized with ethanol to obtain pure products **9a**-**n**.

##### N-(4-cinnamoylphenyl)-1,3-diphenyl-1H-pyrazole-4-carboxamide (9a)

Off-white solid, 147 mg (80% yield), m.p.: 160–161 °C, ^1^H NMR (600 MHz, DMSO-*d*_*6*_) *δ* 10.59 (s, 1H), 9.19 (s, 1H), 8.23 (brs, 2H), 8.05 – 7.84 (m, 9H), 7.76 (d, *J* = 14.3 Hz, 1H), 7.65 – 7.35 (m, 9H).^13^C NMR (150 MHz, DMSO-*d*_*6*_) *δ* 187.6, 161.8, 143.6, 143.4, 138.9, 134.8, 132.5, 132.1, 130.9, 130.5, 129.8, 129.7, 128.9, 128.8, 128.4, 128.2, 128.1, 127.1, 122.0, 119.0, 118.7, 117.5. HRMS (APCI): m/z calcd for C_31_H_24_N_3_O_2_^+^ 470.1863 [M + H]^+^, found 470.1890.

##### (E)-1,3-diphenyl-N-(4-(3-(o-tolyl)acryloyl)phenyl)-1H-pyrazole-4-carboxamide (9b)

Off-white solid, 150 mg (79% yield), m.p.: 180–181 °C, ^1^H NMR (600 MHz, DMSO-*d*_*6*_) *δ* 10.57 (s, 1H), 9.16 (s, 1H), 8.18 (d, *J* = 6.7 Hz, 2H), 8.01 – 7.94 (m, 4H), 7.93 – 7.87 (m, 2H), 7.86 – 7.80 (m, 3H), 7.64 – 7.56 (m, 2H), 7.48 – 7.40 (m, 4H), 7.39 – 7.25 (m, 3H), 2.44 (s, 3H). ^13^C NMR (150 MHz, DMSO-*d*_*6*_) *δ* 187.7, 166.8, 151.4, 143.7, 140.5, 138.0, 137.9, 133.4, 132.5, 132.1, 130.9, 130.8, 130.3, 129.9, 129.8, 128.5, 128.3, 128.2, 127.2, 126.8, 126.4, 122.9, 119.1, 118.8, 117.5, 19.3. HRMS (APCI): m/z calcd for C_32_H_26_N_3_O_2_^+^ 484.2020 [M + H]^+^, found 484.2025.

##### (E)-N-(4-(3-(3-methoxyphenyl)acryloyl)phenyl)-1,3-diphenyl-1H-pyrazole-4-carboxamide (9c)

Off-white solid, 161 mg (82% yield), m.p.: 186–187 °C, ^1^H NMR (600 MHz, DMSO-*d*_*6*_) *δ* 10.58 (s, 1H), 9.16 (s, 1H), 8.20 (d, *J* = 7.4 Hz, 2H), 7.96 – 7.90 (m, 5H), 7.85 (d, *J* = 6.0 Hz, 2H), 7.70 (d, *J* = 15.6 Hz, 1H), 7.59 (s, 2H), 7.48 – 7.39 (m, 7H), 7.03 (d, *J* = 7.4 Hz, 1H), 3.84 (s, 3H).^13^C NMR (150 MHz, DMSO-*d*_*6*_) *δ* 187.6, 161.8, 159.7, 143.7, 143.4, 138.9, 136.2, 132.5, 132.1, 130.9, 129.9, 129.9, 129.8, 128.5, 128.3, 128.2, 127.2, 122.3, 121.6, 119.0, 118.8, 117.5, 116.6, 113.4, 55.32. HRMS (APCI): m/z calcd for C_32_H_26_N_3_O_3_^+^ 500.1969 [M + H]^+^, found 500.1989.

##### (E)-N-(4-(3-(4-methoxyphenyl)acryloyl)phenyl)-1,3-diphenyl-1H-pyrazole-4-carboxamide (9d)

Off-white solid, 157 mg (81% yield), m.p.: 174–175 °C, ^1^H NMR (600 MHz, DMSO-*d*_*6*_) *δ* 10.55 (s, 1H), 9.17 (s, 1H), 8.18 (d, *J* = 8.3 Hz, 2H), 7.96 (d, *J* = 7.9 Hz, 2H), 7.92 – 7.80 (m, 7H), 7.71 (d, *J* = 15.4 Hz, 1H), 7.59 (t, *J* = 7.6 Hz, 2H), 7.48 – 7.39 (m, 4H), 7.03 (d, *J* = 8.3 Hz, 2H), 3.83 (s, 3H).^13^C NMR (150 MHz, DMSO-*d*_*6*_) *δ* 187.4, 161.7, 161.2, 151.3, 143.4, 143.3, 138.9, 132.7, 132.0, 130.8, 130.6, 129.7, 129.6, 128.4, 128.2, 128.1, 127.4, 127.1, 119.4, 118.9, 118.7, 117.5, 114.3, 55.3. HRMS (APCI): m/z calcd for C_32_H_26_N_3_O_3_^+^ 500.1969 [M + H]^+^, found 500.1990.

##### (E)-N-(4-(3-(4-bromophenyl)acryloyl)phenyl)-1,3-diphenyl-1H-pyrazole-4-carboxamide (9e)

Off-white solid, 167 mg (78% yield), m.p.: 182–183 °C, ^1^H NMR (600 MHz, DMSO-*d*_*6*_) *δ* 10.55 (s, 1H), 9.15 (s, 1H), 8.19 (d, *J* = 8.0 Hz, 2H), 7.96 (d, *J* = 8.3 Hz, 2H), 7.89 (d, *J* = 8.2 Hz, 2H), 7.87 – 7.77 (m, 2H), 7.76 – 7.69 (m, 3H), 7.67 (d, *J* = 7.8 Hz, 3H), 7.59 (s, 2H), 7.48 – 7.40 (m, 3H), 7.36 (d, *J* = 16.1 Hz, 1H).^13^C NMR (150 MHz, DMSO-*d*_*6*_) 187.5, 161.8, 151.4, 143.8, 142.0, 141.6, 139.0, 132.0, 131.9, 130.7, 130.4, 129.9, 129.8, 128.5, 128.3, 128.2, 126.3, 122.8, 119.0, 118.8, 117.5. HRMS (APCI): m/z calcd for C_31_H_23_BrN_3_O_2_^+^ 548.0968 [M + H]^+^, found 548.0968.

##### (E)-N-(4-(3-(2-chlorophenyl)acryloyl)phenyl)-1,3-diphenyl-1H-pyrazole-4-carboxamide (9f)

Off-white solid, 164 mg (83% yield), m.p.: 202–203 °C, ^1^H NMR (600 MHz, DMSO-*d*_*6*_) *δ* 10.58 (s, 1H), 9.18 (s, 1H), 8.23 (d, *J* = 8.1 Hz, 3H), 8.09 – 7.99 (m, 2H), 7.98 – 7.90 (m, 4H), 7.87 (d, *J* = 7.3 Hz, 2H), 7.65 – 7.58 (m, 3H), 7.51 – 7.41 (m, 6H).^13^C NMR (150 MHz, DMSO-*d*_*6*_) *δ* 187.4, 161.8, 151.4, 143.9, 138.9, 138.0, 132.4, 132.2, 131.9, 130.9, 130.0, 129.7, 128.5, 128.4, 128.3, 128.1, 127.7, 127.2, 124.8, 119.0, 118.7, 117.5. HRMS (APCI): m/z calcd for C_31_H_23_ClN_3_O_2_^+^ 504.1473 [M + H]^+^, found 504.1445.

##### (E)-N-(4-(3-(3-chlorophenyl)acryloyl)phenyl)-1,3-diphenyl-1H-pyrazole-4-carboxamide (9 g)

Off-white solid, 152 mg (77% yield), m.p.: 167–168 °C, ^1^H NMR (600 MHz, DMSO-*d*_*6*_) *δ* 10.56 (s, 1H), 9.17 (s, 1H), 8.22 (s, 2H), 8.15 – 8.03 (m, 2H), 8.00 – 7.77 (m, 7H), 7.71 (d, *J* = 15.3 Hz, 1H), 7.59 (s, 2H), 7.55 – 7.23 (m, 6H).^13^C NMR (150 MHz, DMSO-*d*_*6*_) *δ* 187.4, 161.8, 151.4, 143.8, 141.6, 138.9, 137.0, 130.9, 130.7, 130.0, 129.7, 128.4, 128.3, 128.1, 127.8, 127.8, 123.5, 119.0, 118.7, 117.4. HRMS (APCI): m/z calcd for C_31_H_23_ClN_3_O_2_^+^ 504.1473 [M + H]^+^, found 504.1468.

##### (E)-N-(4-(3-(3-fluorophenyl)acryloyl)phenyl)-1,3-diphenyl-1H-pyrazole-4-carboxamide (9 h)

Off-white solid, 145 mg (76% yield), m.p.: 138–139 °C, ^1^H NMR (600 MHz, DMSO-*d*_*6*_) *δ* 10.60 (s, 1H), 9.20 (s, 1H), 8.22 (s, 2H), 8.08 – 7.80 (m, 9H), 7.71 (brs, 2H), 7.65 – 7.40 (m, 6H), 7.29 (s, 1H).^13^C NMR (150 MHz, DMSO-*d*_*6*_) *δ* 191.6, 161.8, 152.4, 143.8, 141.9, 131.0, 129.9, 129.7 (d, *J*_C−F_ = 27 Hz), 128.4, 128.3, 128.1 (d, *J*_C−F_ = 21 Hz), 127.2, 125.4, 123.5, 123.3 (d, *J*_C−F_ = 3 Hz), 119.0, 118.7, 117.4, 114.6, 114.5 (d, *J*_C−F_ = 18 Hz). HRMS (APCI): m/z calcd for C_31_H_23_FN_3_O_2_^+^ 488.1769 [M + H]^+^, found 488.1767.

##### (E)-N-(4-(3-(4-fluorophenyl)acryloyl)phenyl)-1,3-diphenyl-1H-pyrazole-4-carboxamide (9i)

Off-white solid, 155 mg (81% yield), m.p.: 212–213 °C, ^1^H NMR (600 MHz, DMSO-*d*_*6*_) *δ* 10.55 (s, 1H), 9.16 (s, 1H), 8.20 (s, 2H), 8.05 – 7.83 (m, 9H), 7.74 (d, *J* = 14.8 Hz, 1H), 7.68 – 7.22 (m, 8H).^13^C NMR (150 MHz, DMSO-*d*_*6*_) *δ* 187.5, 161.8, 151.4, 143.7, 142.1, 138.9, 132.1 (d, *J*_C−F_ = 57 Hz), 131.1 (d, *J*_C−F_ = 9 Hz), 130.9, 129.8, 129.7 (d, *J*_C−F_ = 13.5 Hz), 128.4, 128.3 (d, *J*_C−F_ = 30 Hz), 128.1, 127.2, 121.9, 119.0, 118.7, 117.5, 116.0, 115.8 (d, *J*_C−F_ = 22.5 Hz). HRMS (APCI): m/z calcd for C_31_H_23_FN_3_O_2_^+^ 488.1769 [M + H]^+^, found 488.1759.

##### (E)-N-(4-(3-(3,4-dichlorophenyl)acryloyl)phenyl)-1,3-diphenyl-1H-pyrazole-4-carboxamide (9j)

Off-white solid, 173 mg (82% yield), m.p.: 200–201 °C, ^1^H NMR (600 MHz, DMSO-*d*_*6*_) *δ* 10.55 (s, 1H), 9.16 (s, 1H), 8.30 – 8.14 (m, 2H), 8.07 (d, *J* = 15.4 Hz, 1H), 7.96 – 7.84 (m, 7H), 7.65 – 7.60 (m, 2H), 7.58 (s, 2H), 7.48 – 7.36 (m, 5H).^13^C NMR (150 MHz, DMSO-*d*_*6*_) *δ* 187.3, 161.8, 151.4, 140.5, 138.9, 135.7, 130.9, 130.0, 129.7, 129.0, 128.2, 128.1, 124.1, 118.9, 118.7. HRMS (APCI): m/z calcd for C_31_H_22_Cl_2_N_3_O_2_^+^ 538.1084 [M + H]^+^, found 538.1089.

##### (E)-N-(4-(3-(3-nitrophenyl)acryloyl)phenyl)-1,3-diphenyl-1H-pyrazole-4-carboxamide (9 k)

Off-white solid, 167 mg (83% yield), m.p.: 191–192 °C, ^1^H NMR (600 MHz, DMSO-*d*_*6*_) *δ* 10.57 (s, 1H), 9.17 (s, 1H), 8.78 (s, 1H), 8.34 (s, 1H), 8.26 (s, 3H), 8.17 (d, *J* = 15.3 Hz, 1H), 7.97 – 7.84 (m, 7H), 7.76 (s, 1H), 7.59 (s, 2H), 7.48 – 7.36 (m, 4H).^13^C NMR (150 MHz, DMSO-*d*_*6*_) *δ* 187.4, 161.8, 151.4, 143.9, 140.8, 138.9, 136.7, 135.0, 132.1, 130.9, 130.3, 130.1, 129.7, 128.4, 128.3, 128.1, 127.2, 124.8, 124.5, 122.9, 119.0, 118.7. HRMS (APCI): m/z calcd for C_31_H_23_N_4_O_4_^+^ 515.1714 [M + H]^+^, found 515.1722.

##### (E)-1,3-diphenyl-N-(4-(3-(thiophen-2-yl)acryloyl)phenyl)-1H-pyrazole-4-carboxamide (9 l)

Off-white solid, 149 mg (80% yield), m.p.: 210–211 °C, ^1^H NMR (600 MHz, DMSO-*d*_*6*_) *δ* 10.55 (s, 1H), 9.16 (d, *J* = 1.8 Hz, 1H), 8.13 (d, *J* = 4.7 Hz, 2H), 7.97 – 7.83 (m, 7H), 7.78 (s, 1H), 7.68 (s, 1H), 7.58 (d, *J* = 12.0 Hz, 3H), 7.50 – 7.35 (m, 4H), 7.20 (brs, 1H).^13^C NMR (150 MHz, DMSO-*d*_*6*_) *δ* 187.1, 161.8, 151.4, 143.6, 139.8, 138.9, 136.2, 132.6, 132.4, 132.1, 130.9, 130.2, 129.7, 128.7, 128.4, 128.2, 128.1, 127.2, 120.3, 119.1, 118.7, 117.5. HRMS (APCI): m/z calcd for C_29_H_22_SN_3_O_2_^+^ 476.1427 [M + H]^+^, found 476.1428.

##### (E)-1,3-diphenyl-N-(4-(3-(pyridin-3-yl)acryloyl)phenyl)-1H-pyrazole-4-carboxamide (9 m)

Off-white solid, 148 mg (78% yield), m.p.: 198–199 °C, ^1^H NMR (600 MHz, DMSO-*d*_*6*_) *δ* 10.56 (s, 1H), 9.16 (s, 1H), 9.03 (s, 1H), 8.62 (s, 1H), 8.35 (s, 1H), 8.21 (d, *J* = 4.5 Hz, 2H), 8.09 (d, *J* = 15.6 Hz, 1H), 7.98 – 7.82 (m, 6H), 7.76 (d, *J* = 15.8 Hz, 1H), 7.59 (brs, 2H), 7.53 – 7.39 (m, 5H).^13^C NMR (150 MHz, DMSO-*d*_*6*_) *δ* 187.3, 161.8, 151.4, 150.9, 150.2, 143.8, 139.9, 138.9, 135.1, 132.1, 130.9, 130.0, 129.7, 128.4, 128.3, 128.1, 127.2, 123.9, 119.0, 118.7, 117.5. HRMS (APCI): m/z calcd for C_30_H_23_N_4_O_2_^+^ 471.1816 [M + H]^+^, found 471.1821.

##### (E)-N-(4-(3-(benzo[d][1,3]dioxol-5-yl)acryloyl)phenyl)-1,3-diphenyl-1H-pyrazole-4-carboxamide (9n)

Off-white solid, 159 mg (79% yield), m.p.: 182–183 °C, ^1^H NMR (600 MHz, DMSO-*d*_*6*_) *δ* 10.56 (s, 1H), 9.15 (s, 1H), 8.18 (d, *J* = 7.7 Hz, 2H), 7.95 (d, *J* = 7.3 Hz, 2H), 7.88 – 7.83 (m, 3H), 7.69 – 7.64 (m, 2H), 7.59 (t, *J* = 6.7 Hz, 2H), 7.48 – 7.40 (m, 5H), 7.33 (d, *J* = 7.6 Hz, 1H), 6.99 (d, *J* = 7.6 Hz, 2H), 6.09 (s, 2H).^13^C NMR (150 MHz, DMSO-*d*_*6*_) 188.2, 161.8, 151.4, 149.5. 149.4, 148.1, 143.4, 142.4, 140.5, 139.0, 130.9, 129.8, 129.7, 128.5, 128.3, 128.2, 125.2, 123.9, 120.0, 119.0, 118.8, 108.6, 106.6, 101.6. HRMS (APCI): m/z calcd for C_32_H_24_N_3_O_4_^+^ 515.1826 [M + H]^+^, found 515.1840.

### Biology

#### *In vitro* anti-parasitic assay

Antiparasitic assays were performed as described in the earlier literature (Bouton et al. 2021). Briefly, to evaluate anti-*Leishmania* activity, *L. infantum* [MHOM/MA (BE)/67] was used with primary peritoneal mouse macrophages as host cell. 3 × 10^4^ macrophages were infected with 4.5 × 10^5^ parasites per well. Compound dilutions were added after 2 h of infection. After 5 days of incubation, parasite burdens (mean number of amastigotes/macrophage) were assessed microscopically after staining with a 10% Giemsa solution. For *T.* *cruzi*, the Tulahuen CL2, β-galactosidase strain (nifurtimox-sensitive) was used, maintained on MRC-5_SV2_ (human lung fibroblast). 4 × 10^3^ cells were infected with 4 × 10^4^ parasites per well. Parasite burdens were assessed after adding the substrate CPRG (chlorophenolred *β*-d-galactopyranoside). The change in color was measured spectrophotometrically at 540 nm after 4 h incubation at 37 °C. Drug susceptibility tests for *T. b. brucei* were performed using a resazurin assay. Susceptibility assays were performed with *T. b. brucei* Squib 42749 or *T. b. rhodesiense* STIB-90050. *T. b. brucei* Squib 427 was seeded at 1.5 × 10^4^ parasites/well and *T. b. rhodesiense* at 4 × 10^3^ parasites per well, followed by the addition of resazurin after 72 h. Incubation with resazurin was for 24 h (*T. b. brucei*) or 6 h (*T. b. rhodesiense*) followed by fluorescence detection.

In all assays, parasite growth was compared to untreated-infected controls (100% growth) and noninfected controls (0% growth). Results were expressed as % parasite reduction at the different drug concentrations and used to calculate IC_50_ values from the dose–response curves. Three repeats were performed for each experiment and the median value recorded.

#### *In vitro* cytotoxicity assay

MRC-5_SV2_ cell cytotoxicity was evaluated as described in the earlier literature (Bouton et al. 2021). Briefly, 1.5 × 10^5^ cells/mL cells were cultured with compound dilutions at 37 °C and with 5% CO_2_. Cell growth was compared to untreated-control wells (100% cell growth) and medium-control wells (0% cell growth). After 3 days incubation, cell viability was assessed fluorometrically after addition of 50 μL resazurin per well. After 4 h at 37 °C, fluorescence was measured (*λ*_ex_ 550 nm, *λ*_em_ 590 nm). The results were expressed as % reduction in cell growth/viability compared to control wells and an IC_50_ value was determined.

## Supplementary Information

Below is the link to the electronic supplementary material.

Supplementary file1 (DOCX 3162 KB)

Supplementary file2 (DOCX 3163 KB)

## Data Availability

No datasets were generated or analysed during the current study.
