# Supplementary material for: Pyrazolyl amide-chalcones conjugates: Synthesis and antikinetoplastid activity
Source: Naunyn Schmiedebergs Arch Pharmacol. 2024 Oct 21;398(4):4199–210. doi: 10.1007/s00210-024-03524-7 (PMC11978679; doi:10.1007/s00210-024-03524-7)
Supplement: Supplementary file 1 — Supplementary file1 (DOCX 3162 KB) [file 210_2024_3524_MOESM1_ESM.docx]

**Pyrazolyl amide-chalcones conjugates: Synthesis and antikinetoplastid activity**

Devesh S Agarwal*^a^*, Richard M. Beteck*^a^*, Dorien Mabille*^b^*, Guy Caljon*^b^*, Lesetja J. Legoabe*^a^**

*^a^*Centre of Excellence for Pharmaceutical Sciences, North-West University, Potchefstroom 2520, South Africa

*^b^*Laboratory of Microbiology, Parasitology and Hygiene, Infla-med Centre of Excellence, University of Antwerp, Antwerp, Belgium

*Corresponding Author: LJL- lesetja.legoabe@nwu.ac.za; Tel.: +27 18 299 2182.

**Supporting information**

| **S. No.** |  | **Page No.** |
| --- | --- | --- |
| **1** | ^1^H and ^13^C NMR of **4** | 2 |
| **2** | ^1^H and ^13^C NMR of **5** | 3 |
| **3** | ^1^H and ^13^C NMR of **7** | 4 |
| **4** | ^1^H and ^13^C NMR of **9a**-**n** | 5 |
| **5** | HRMS spectra of **4**, **5**, **7** and **9a**-**n** | 19 |

**^1^H NMR of 4**

**
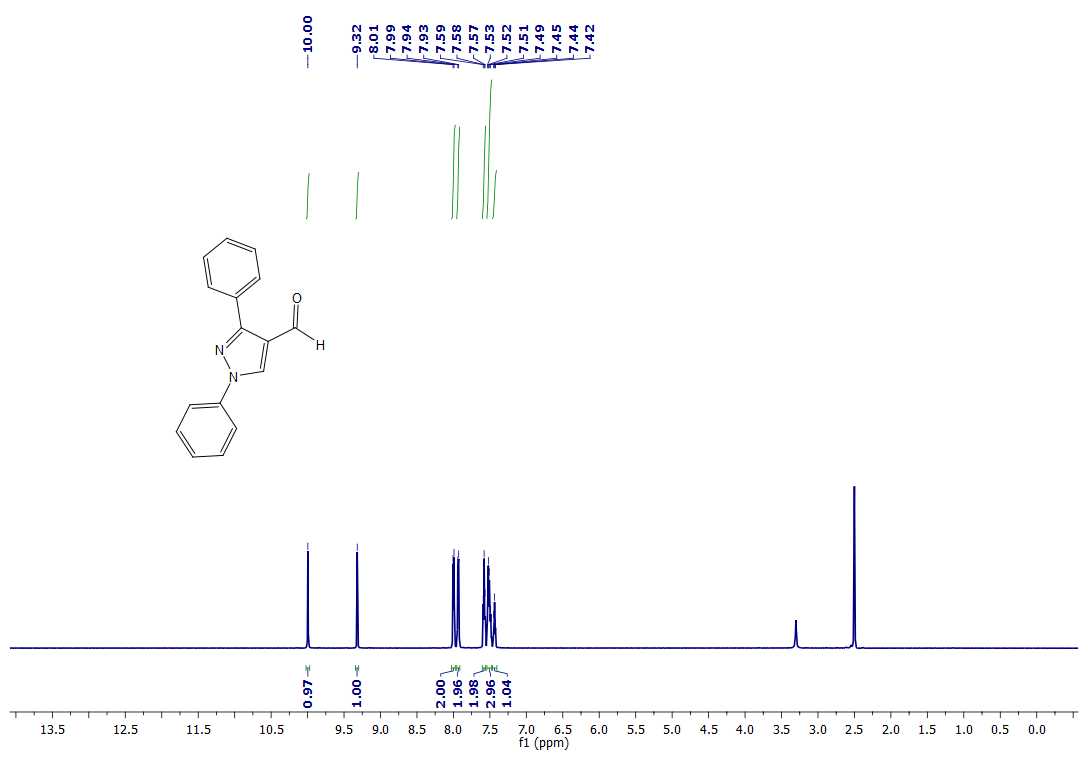
**

**^13^C NMR of 4**

**
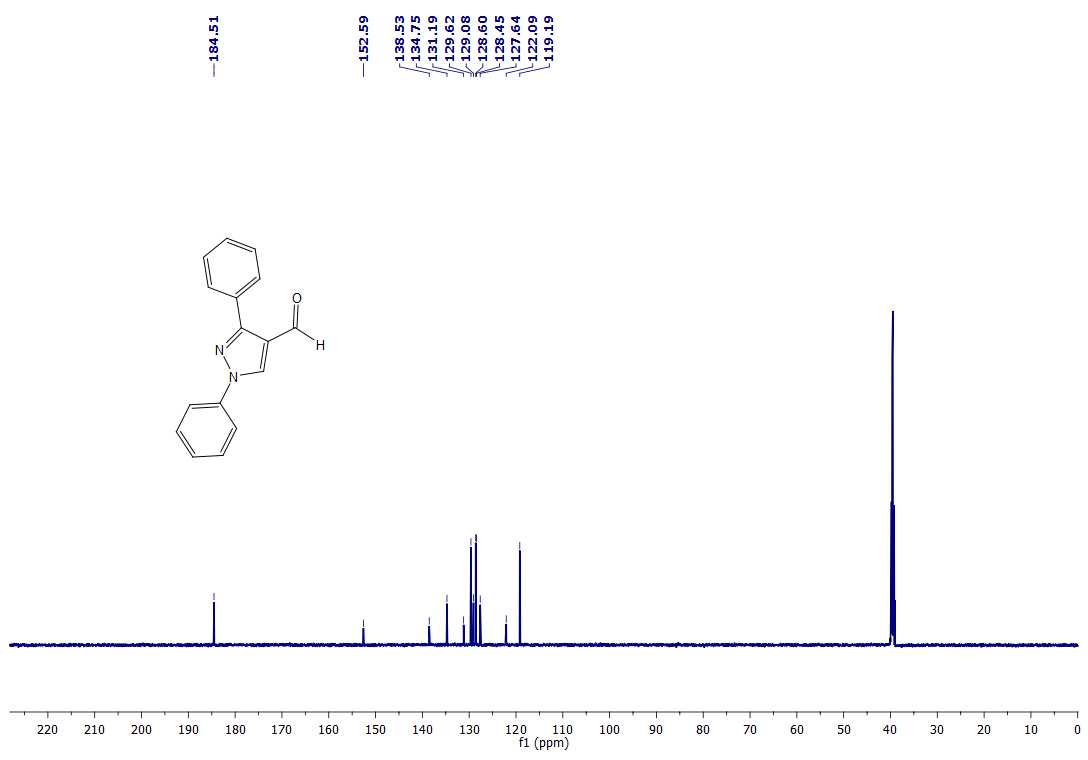
**

**^1^H NMR of 5**

**
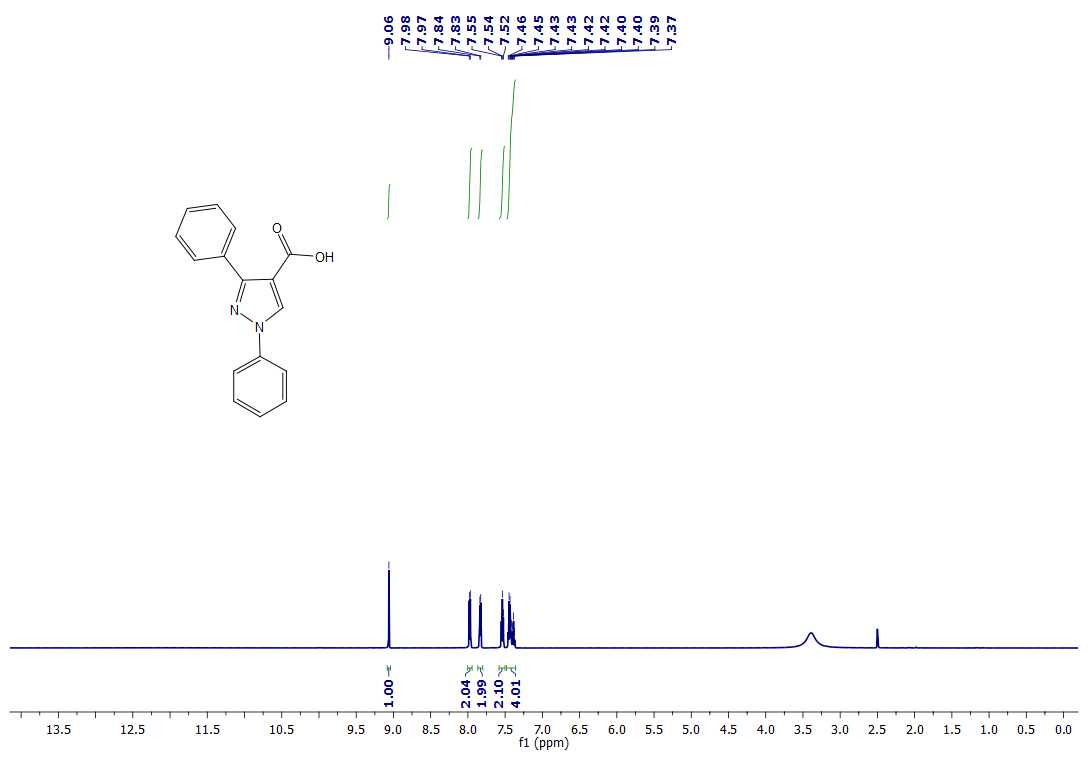
**

**^13^C NMR of 5**

**
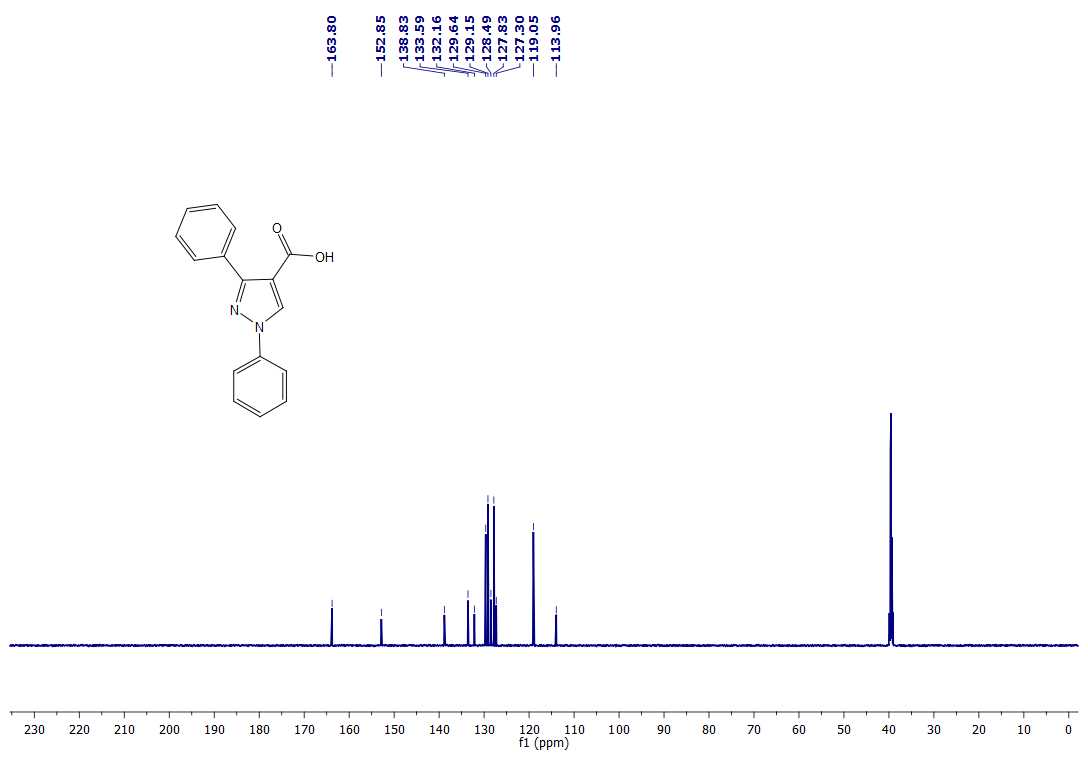
**

**^1^H NMR of 7**

**
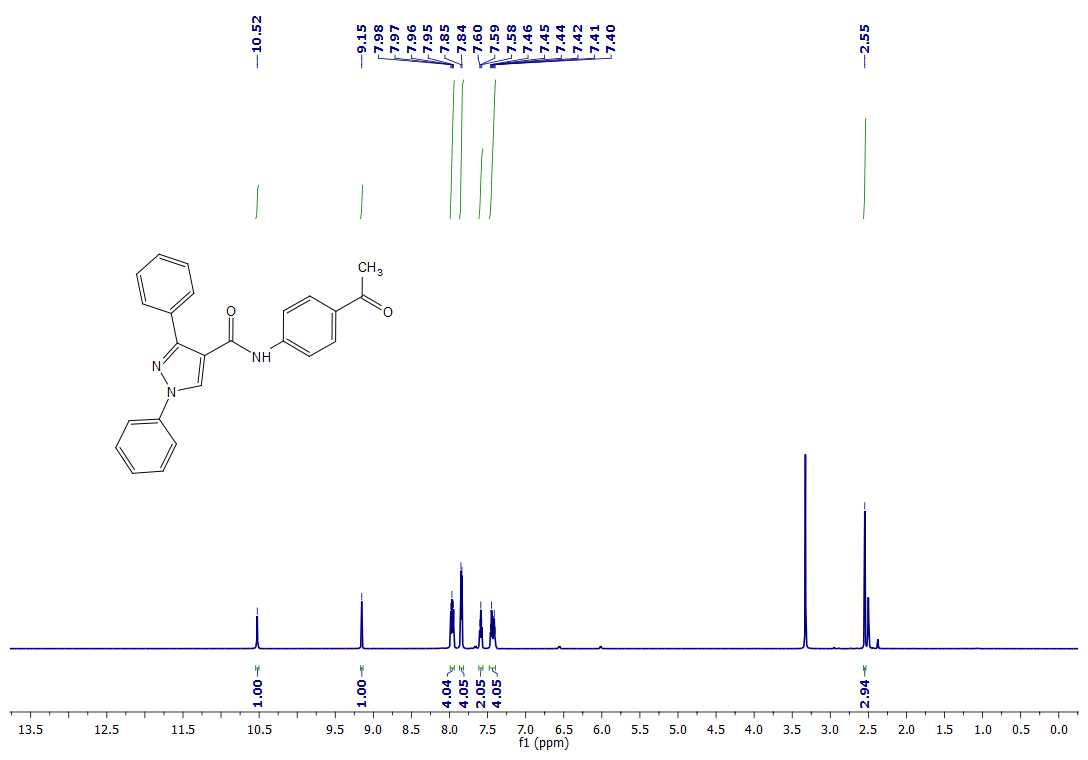
**

**^13^C NMR of 7**

**
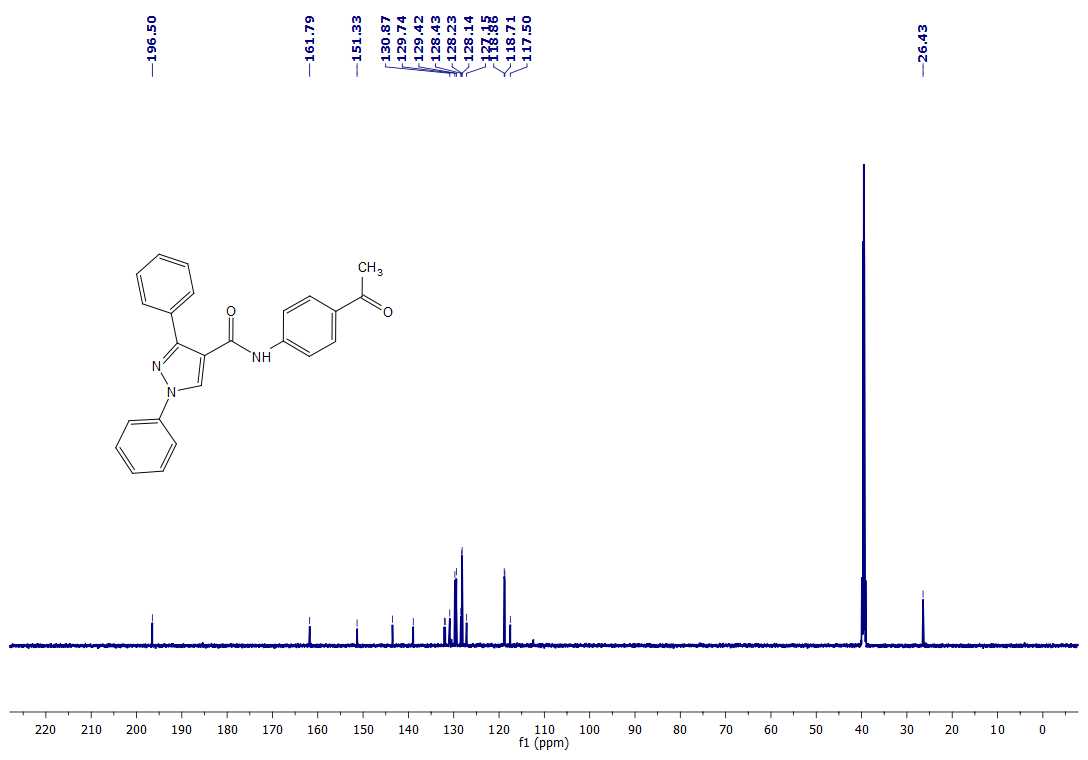
**

**^1^H NMR of 9a**

**
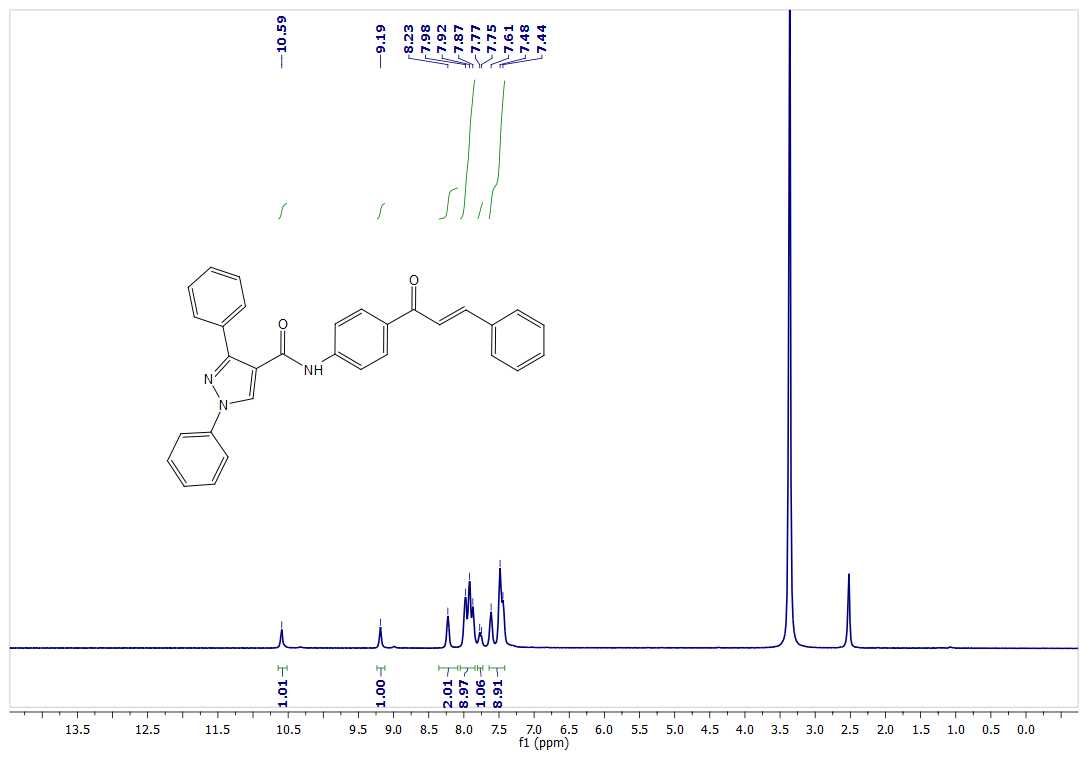
**

**^13^C NMR of 9a**

**
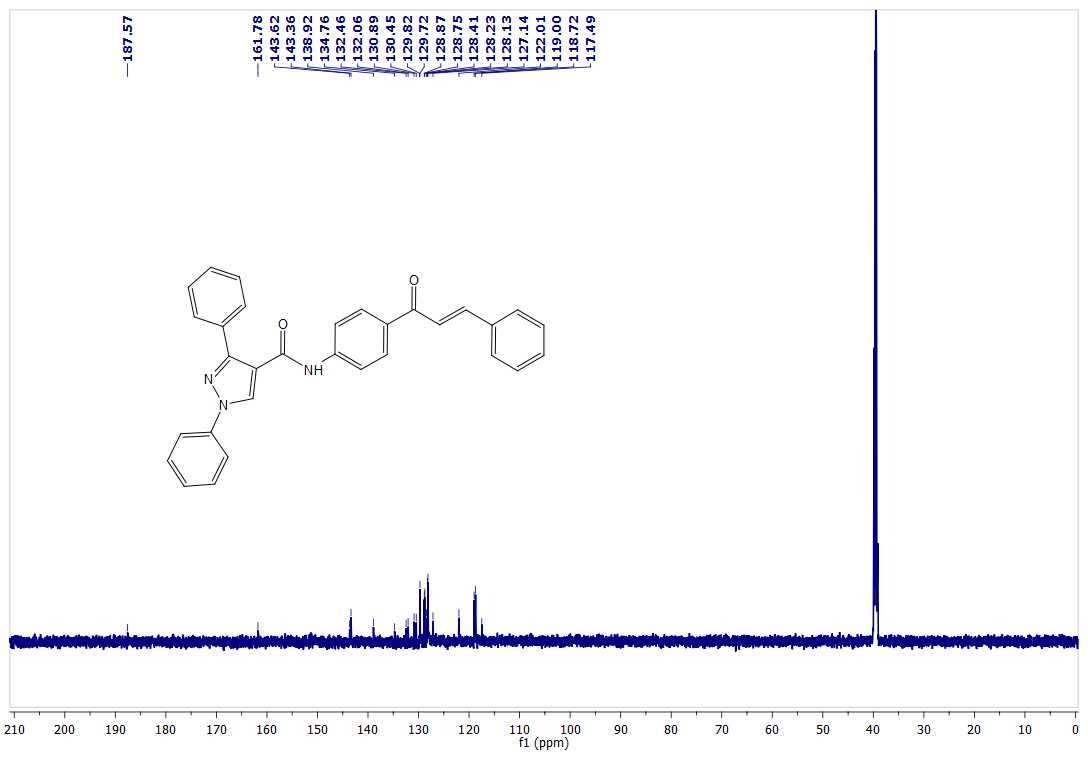
**

**^1^H NMR of 9b**

**
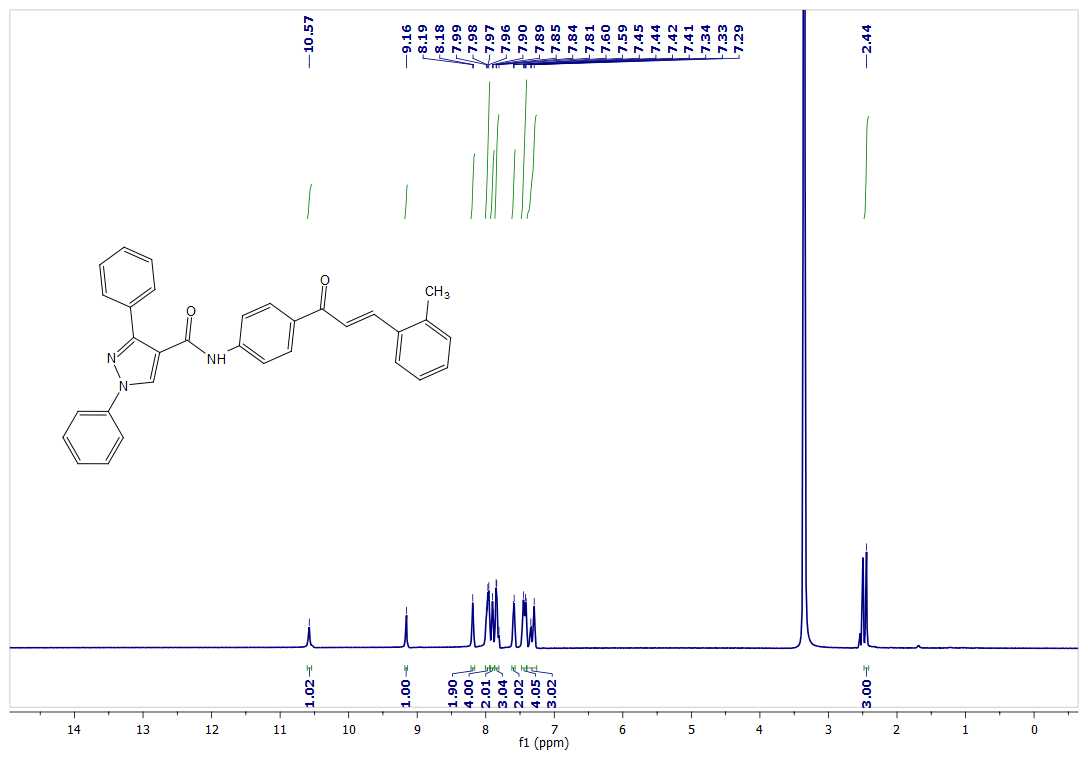
**

**^13^C NMR of 9b**

**
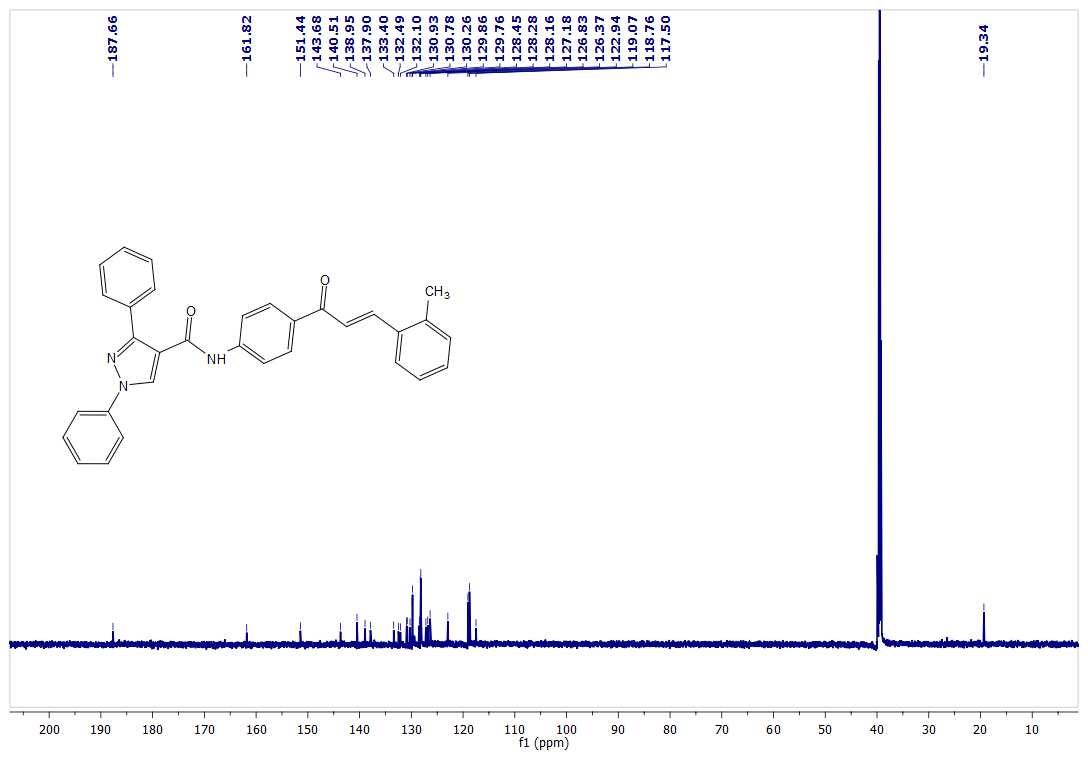
**

**^1^H NMR of 9c**

**
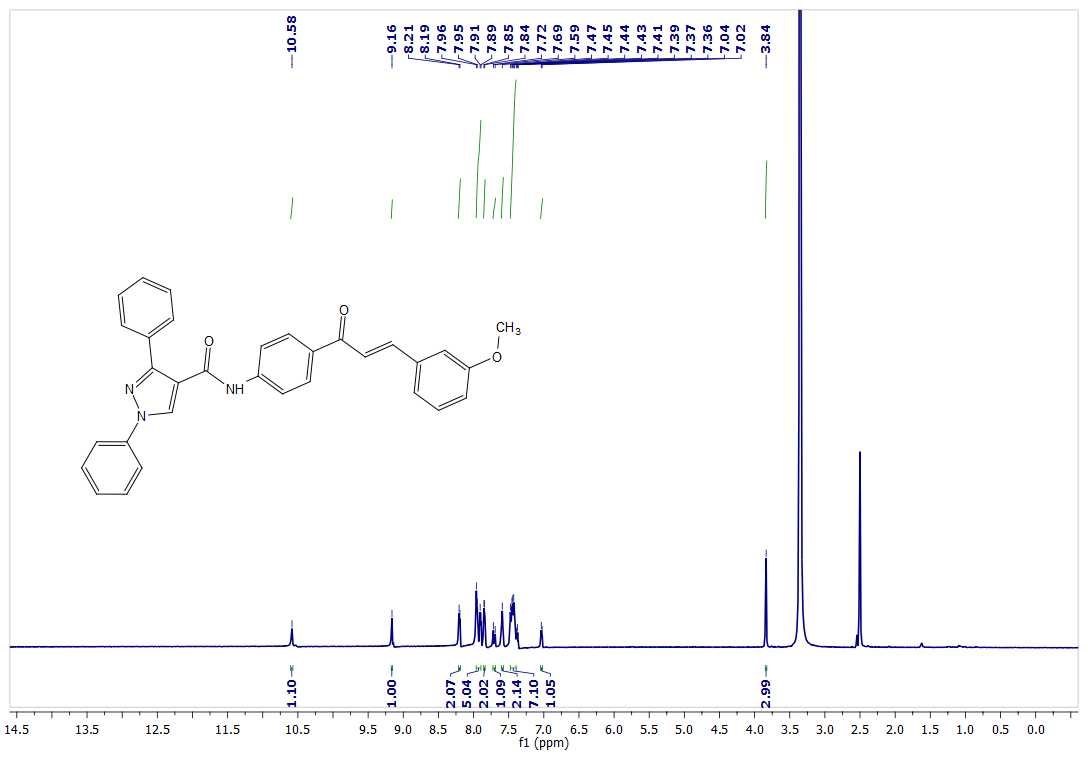
**

**^13^C NMR of 9c**

**
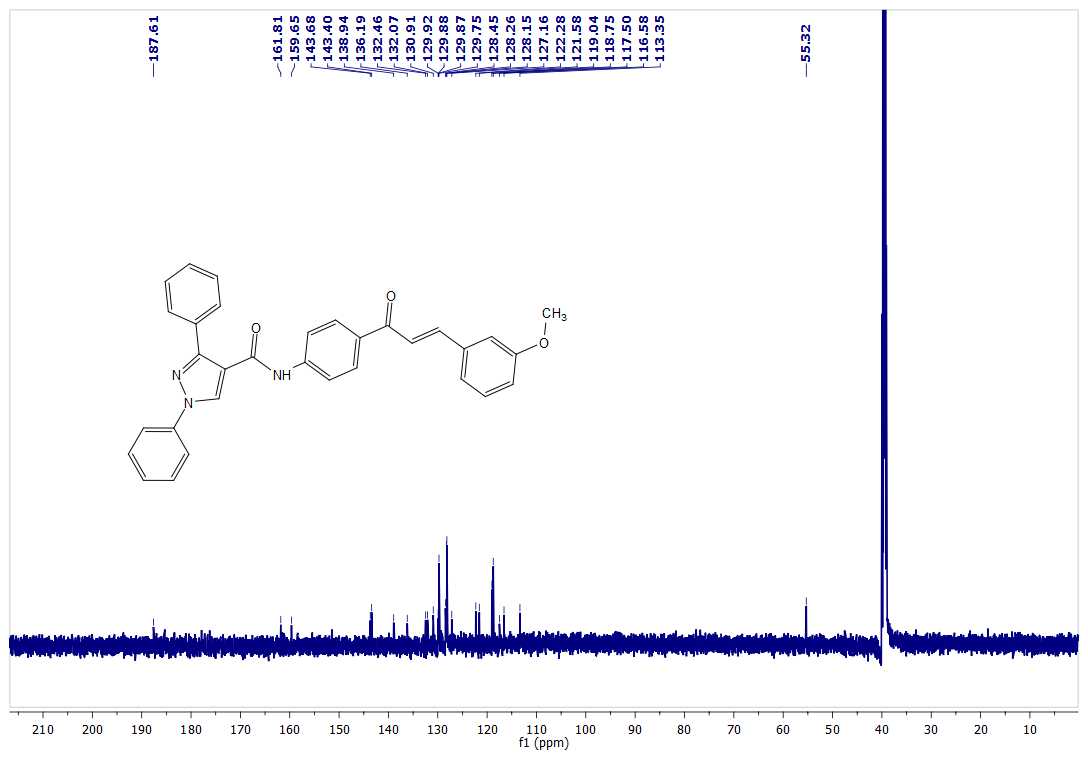
**

**^1^H NMR of 9d**

**
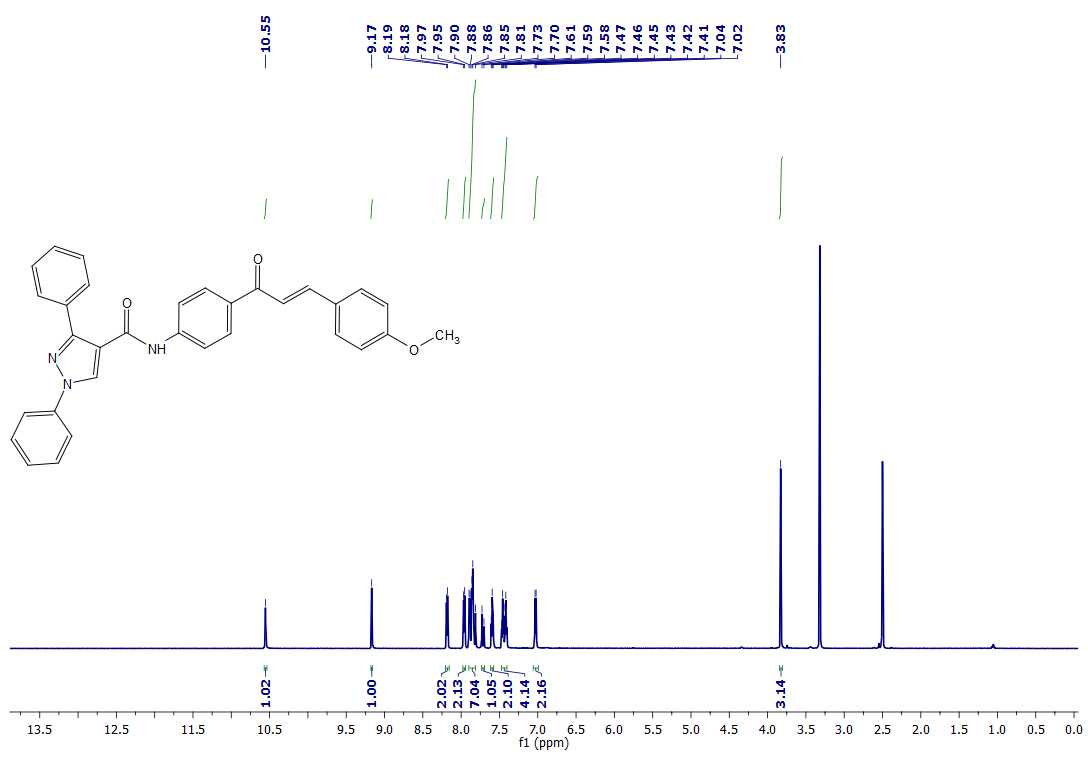
**

**^13^C NMR of 9d**

**
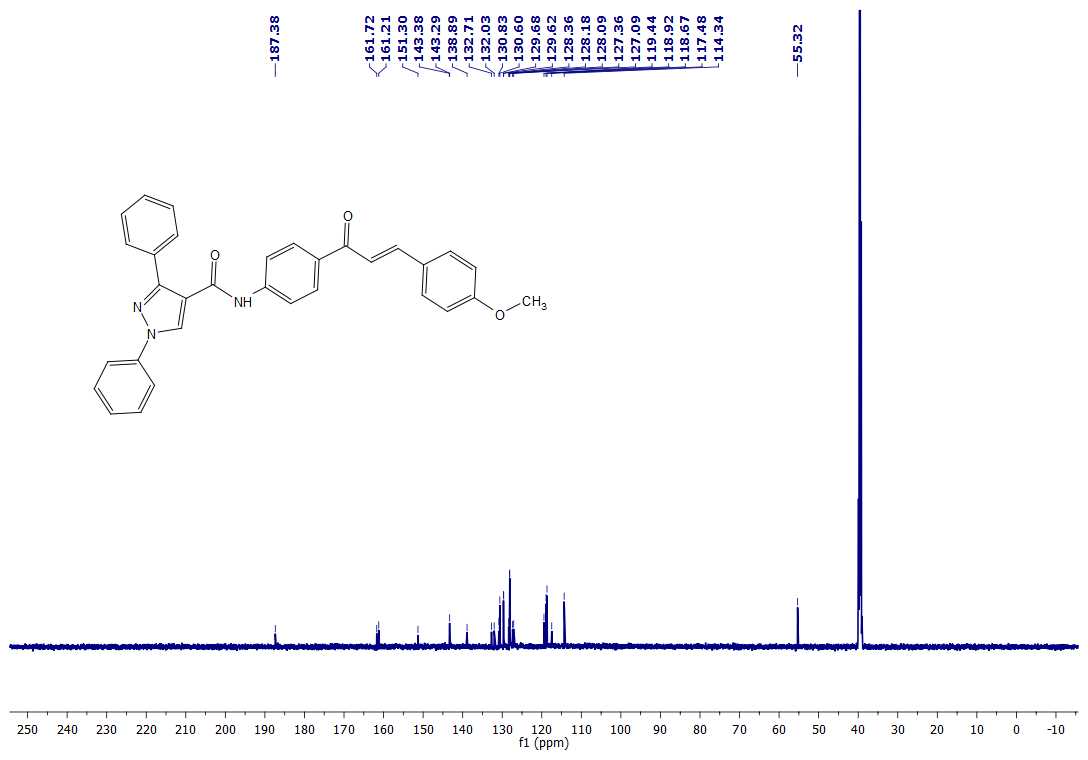
**

**^1^H NMR of 9e**

**
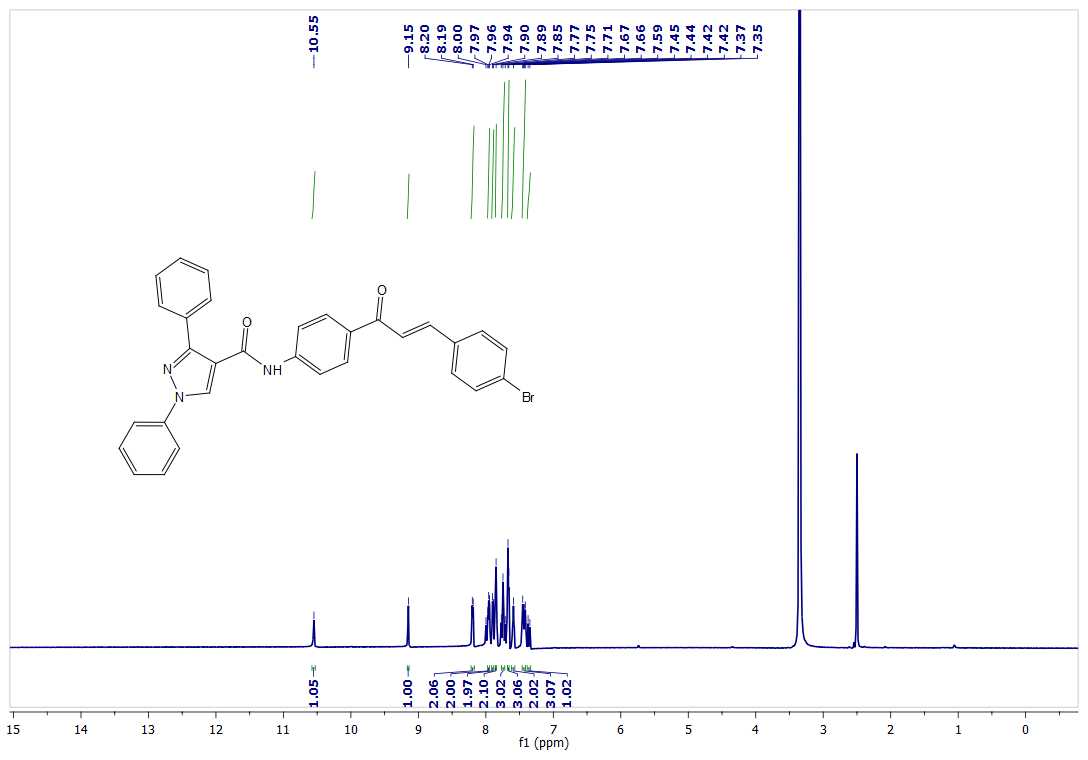
**

**^13^C NMR of 9e**

**
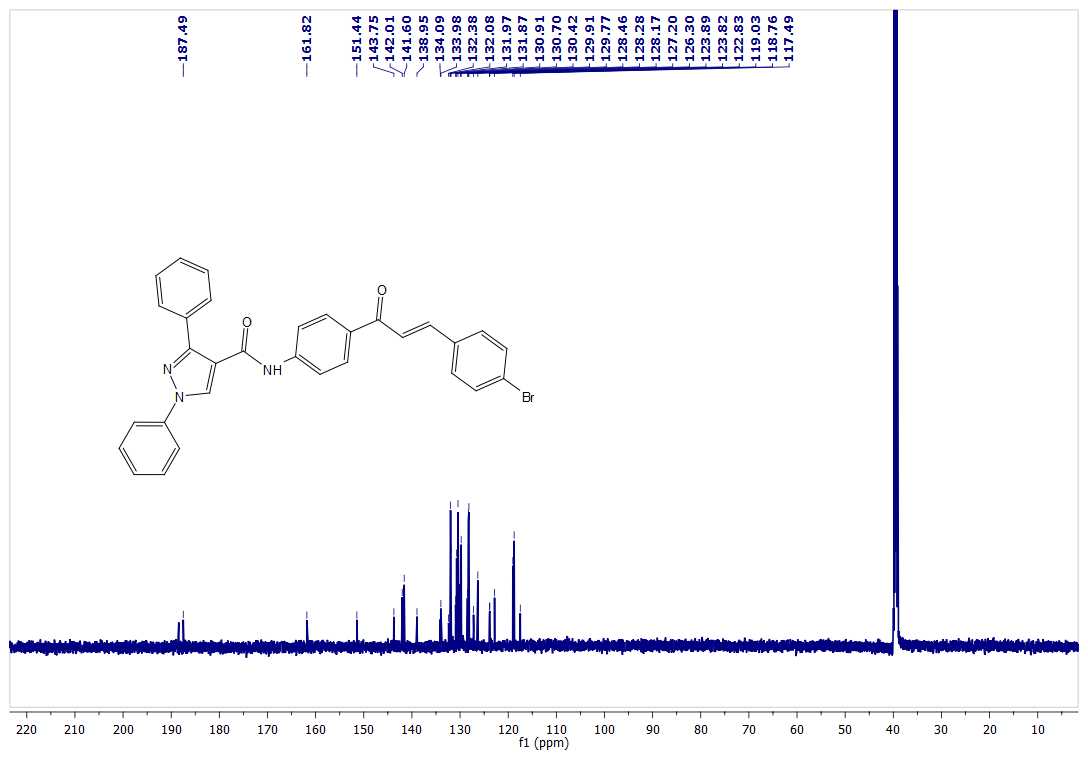
**

**^1^H NMR of 9f**

**
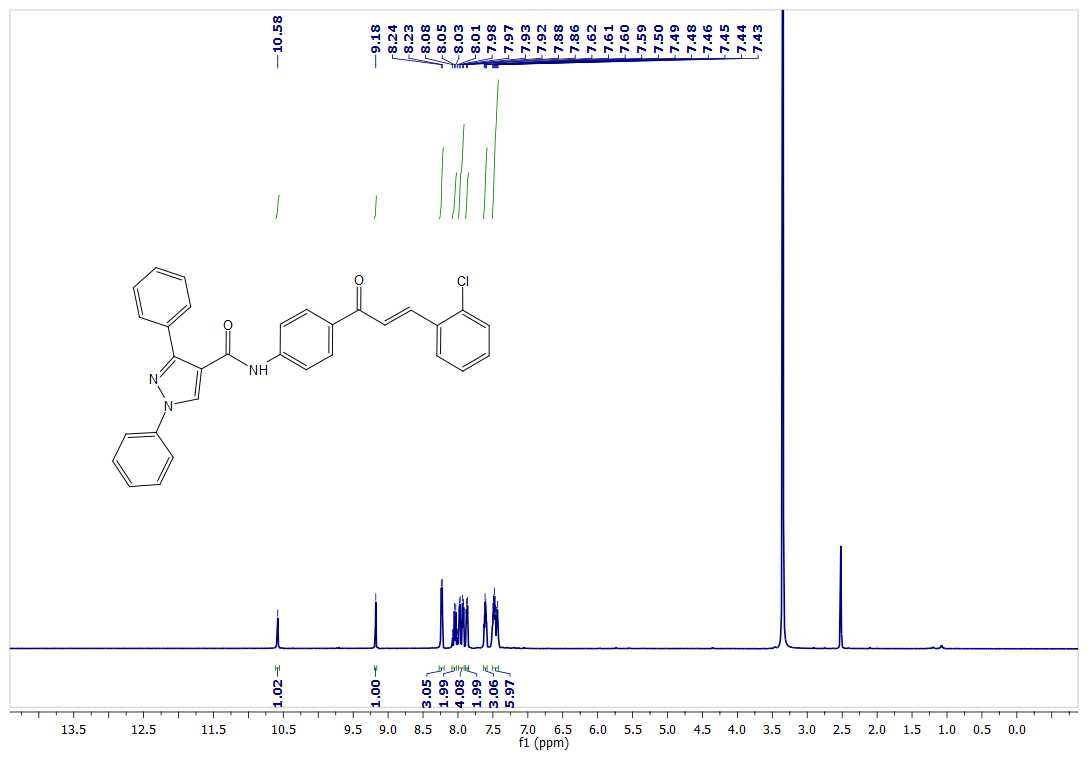
**

**^13^C NMR of 9f**

**^
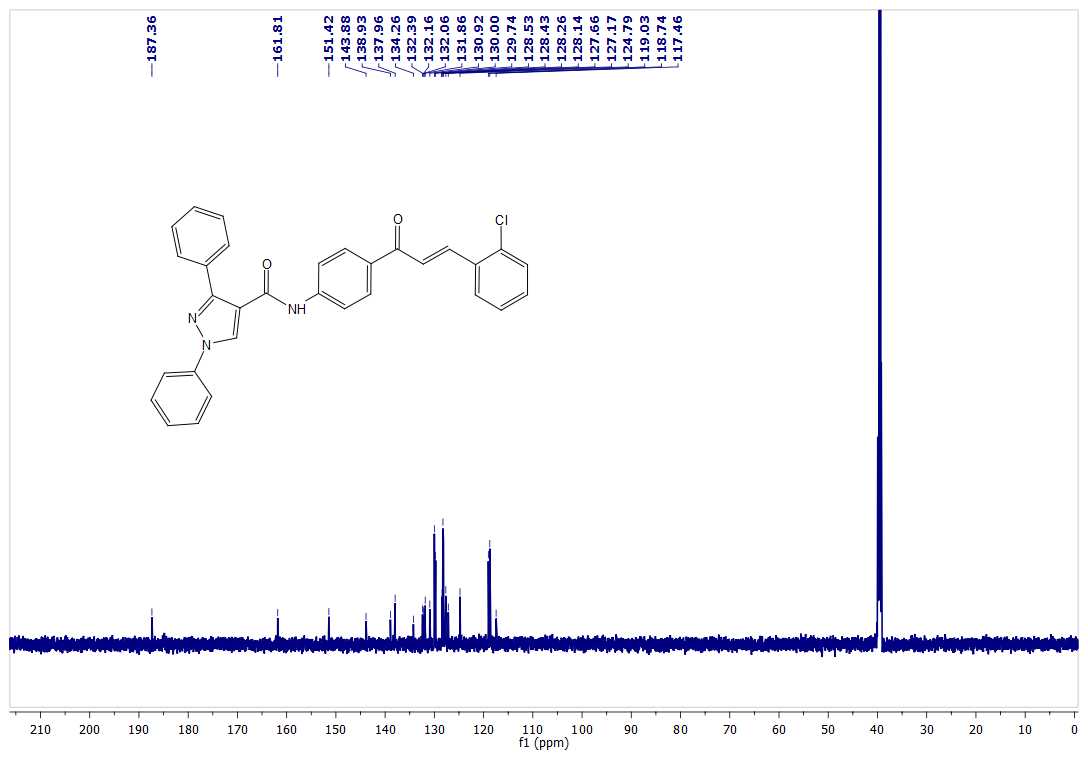
^**

**^1^H NMR of 9g**

**
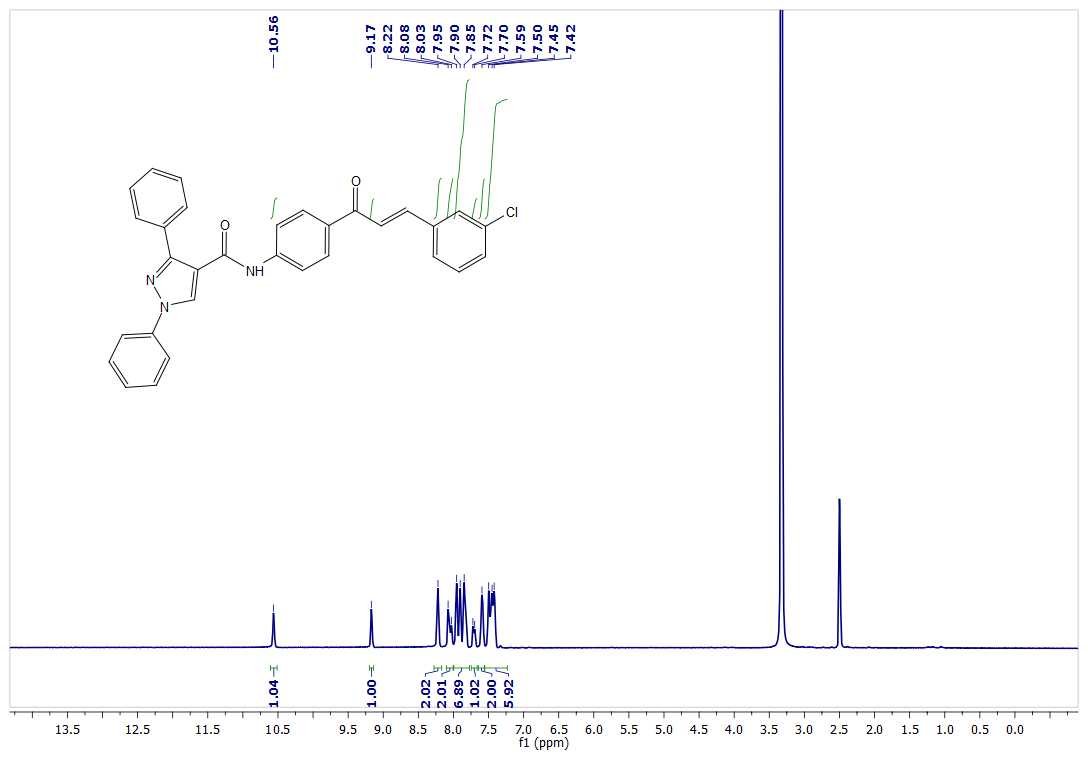
**

**^13^C NMR of 9g**

**
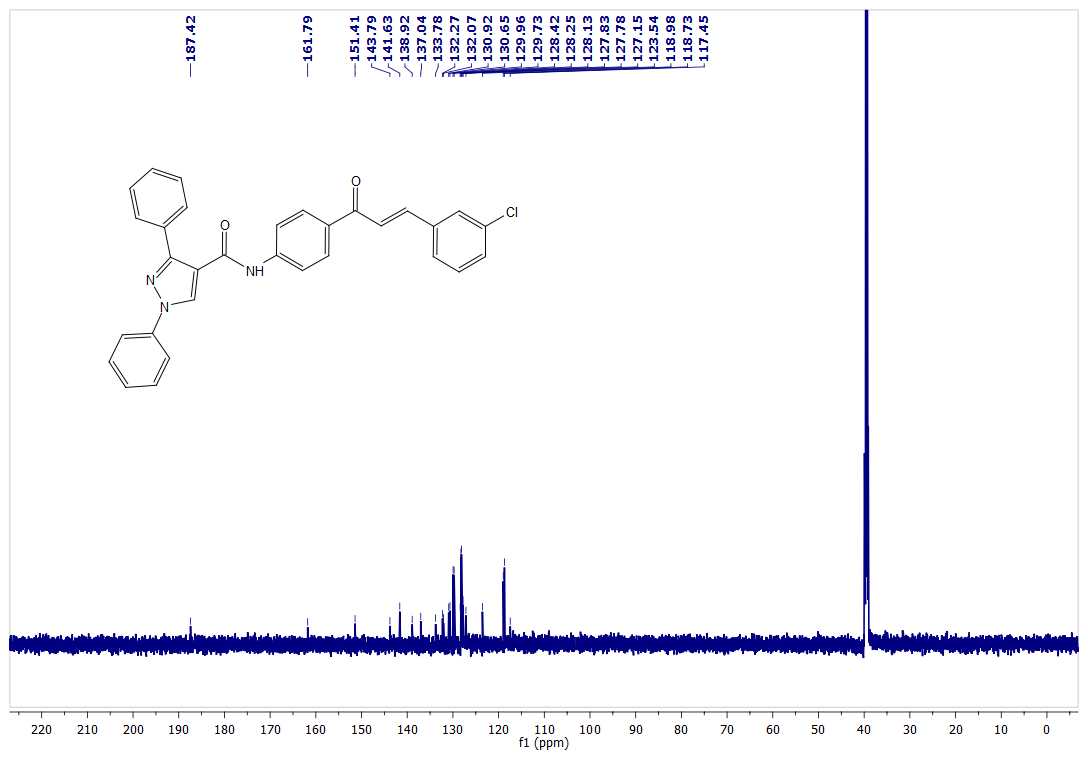
**

**^1^H NMR of 9h**

**
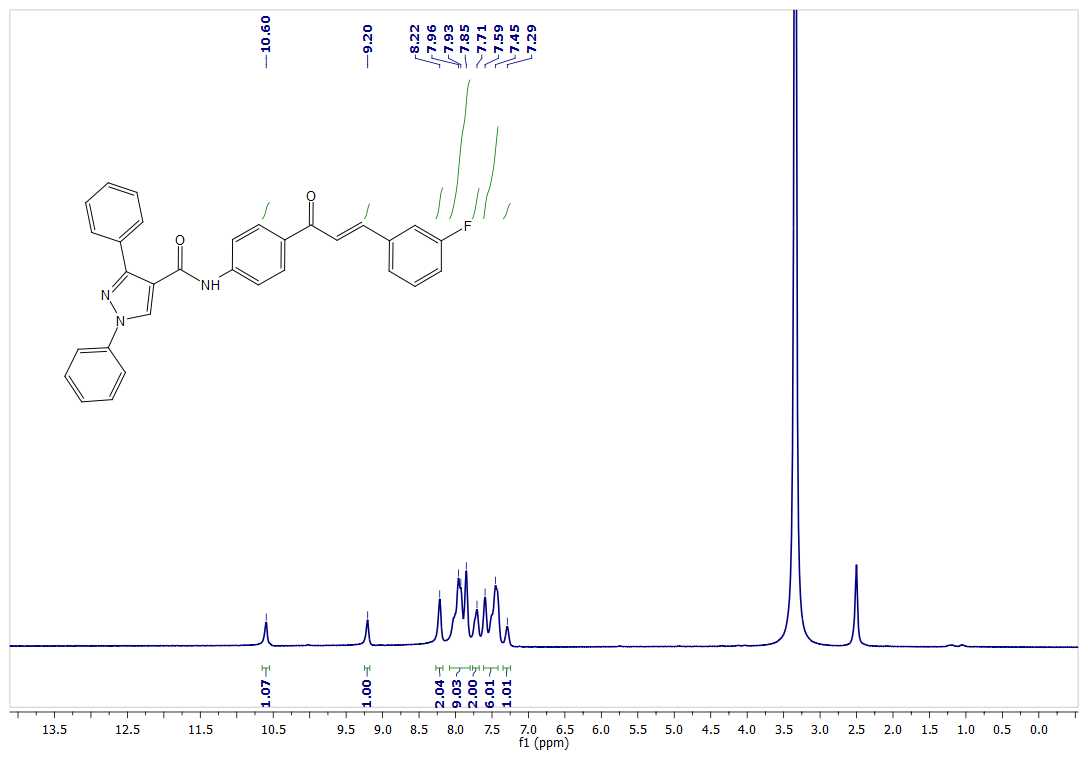
**

**^13^C NMR of 9h**

**
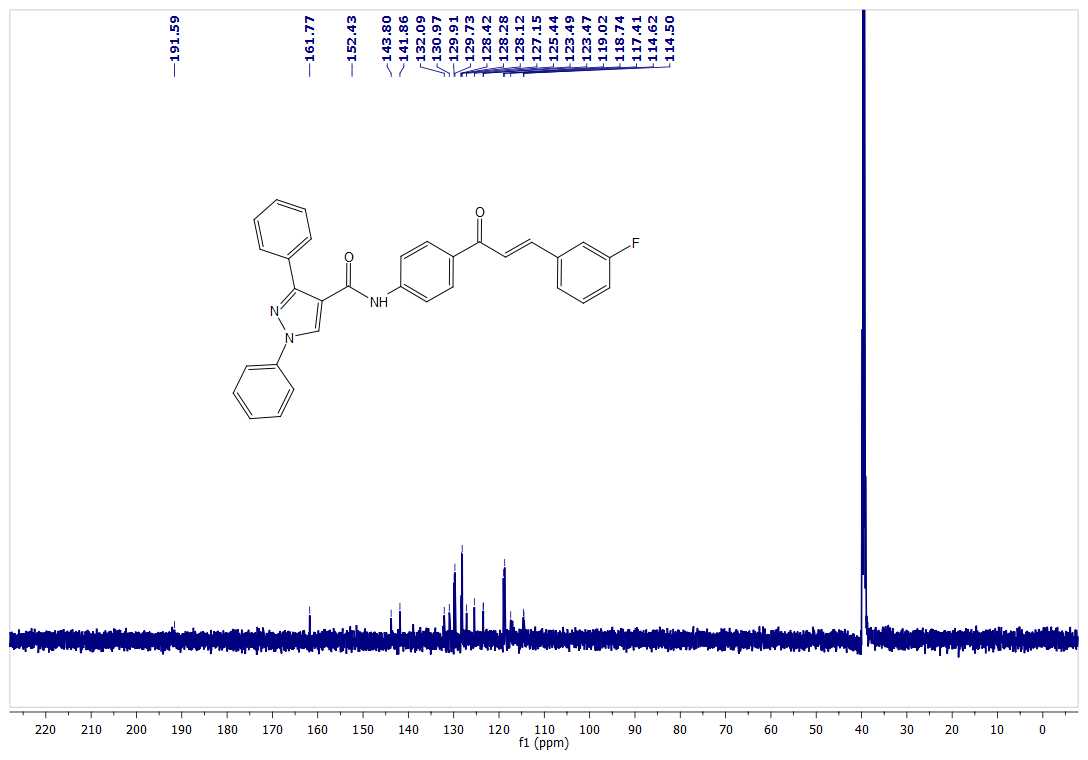
**

**^1^H NMR of 9i**

**
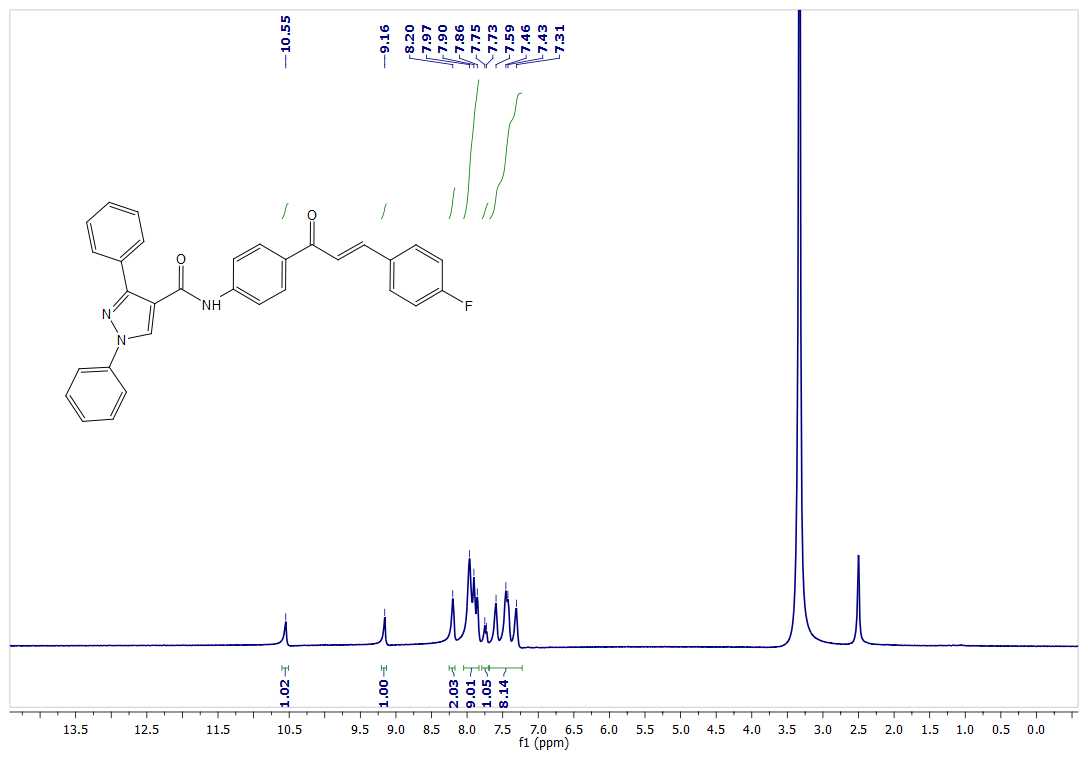
**

**^13^C NMR of 9i**

**
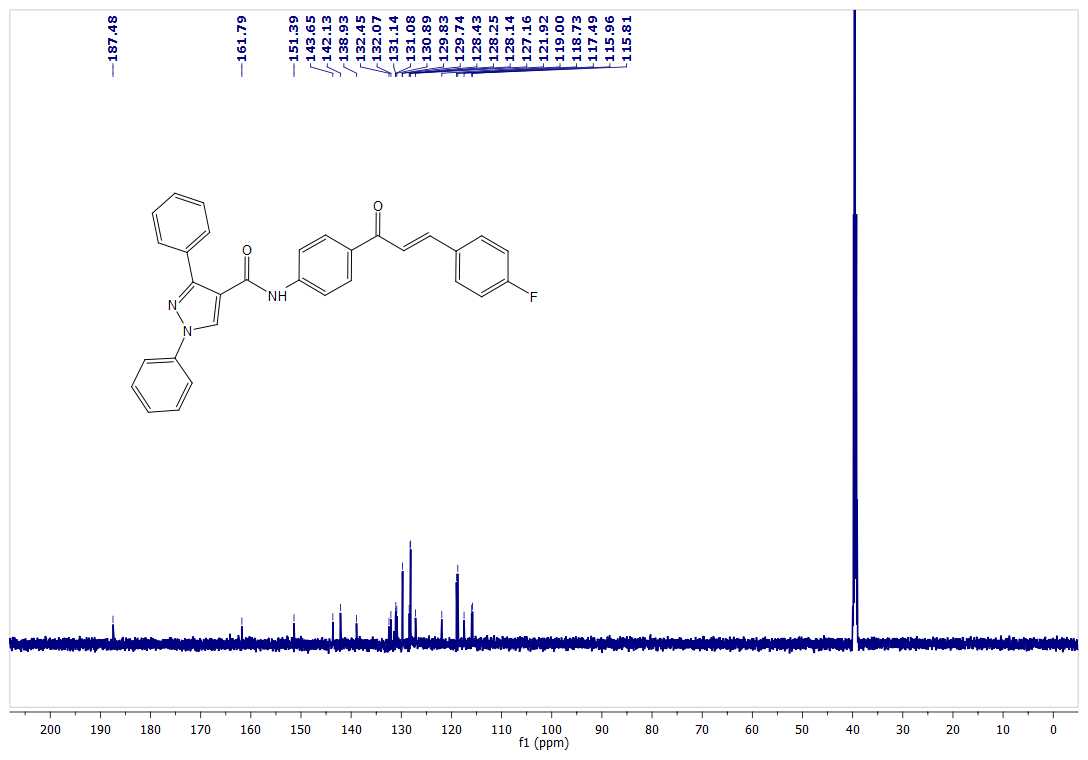
**

**^1^H NMR of 9j**

**
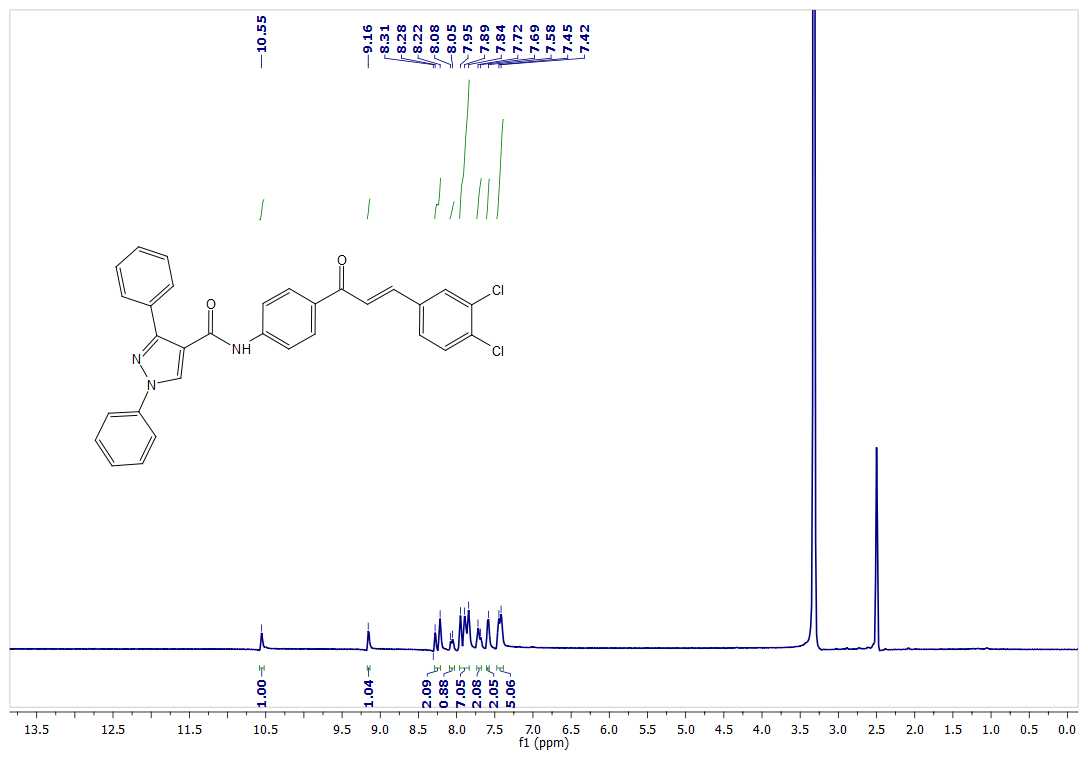
**

**^13^C NMR of 9j**

**
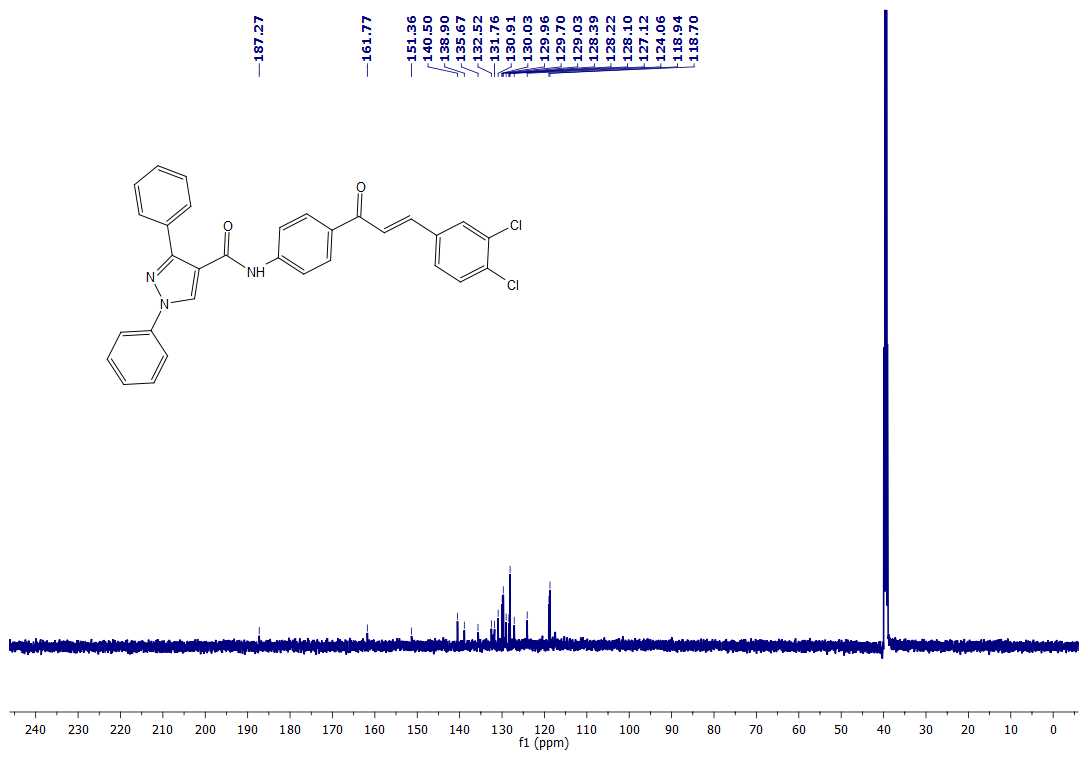
**

**^1^H NMR of 9k**

**
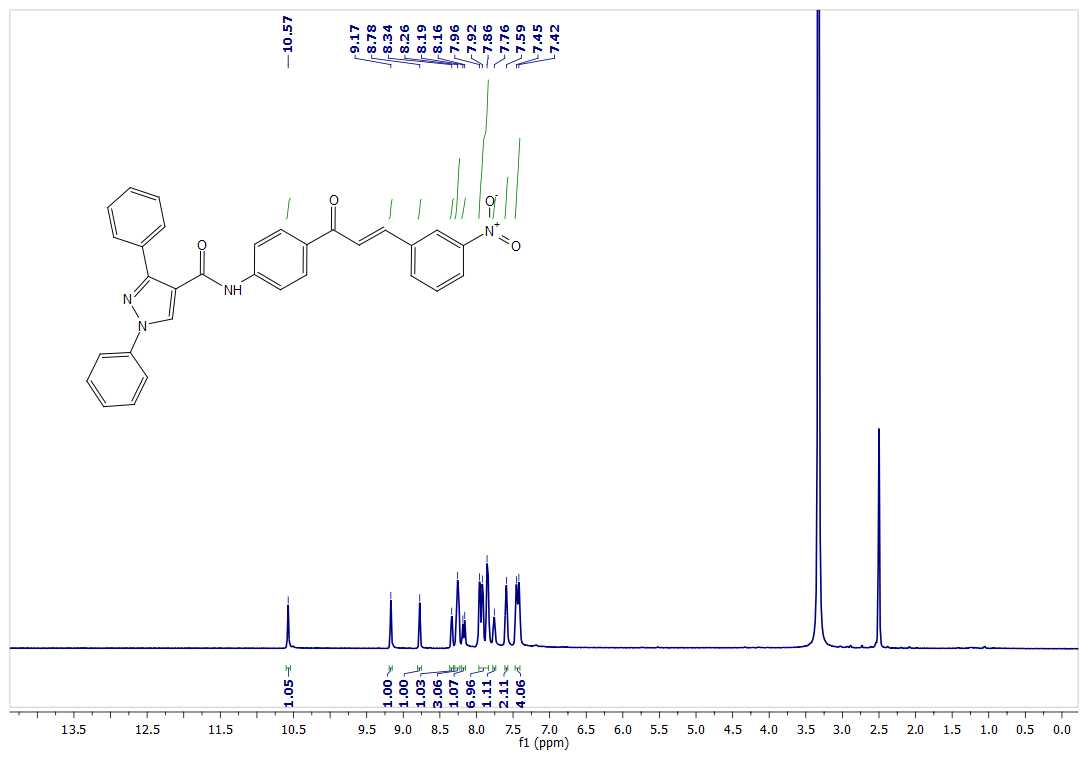
**

**^13^C NMR of 9k**

**
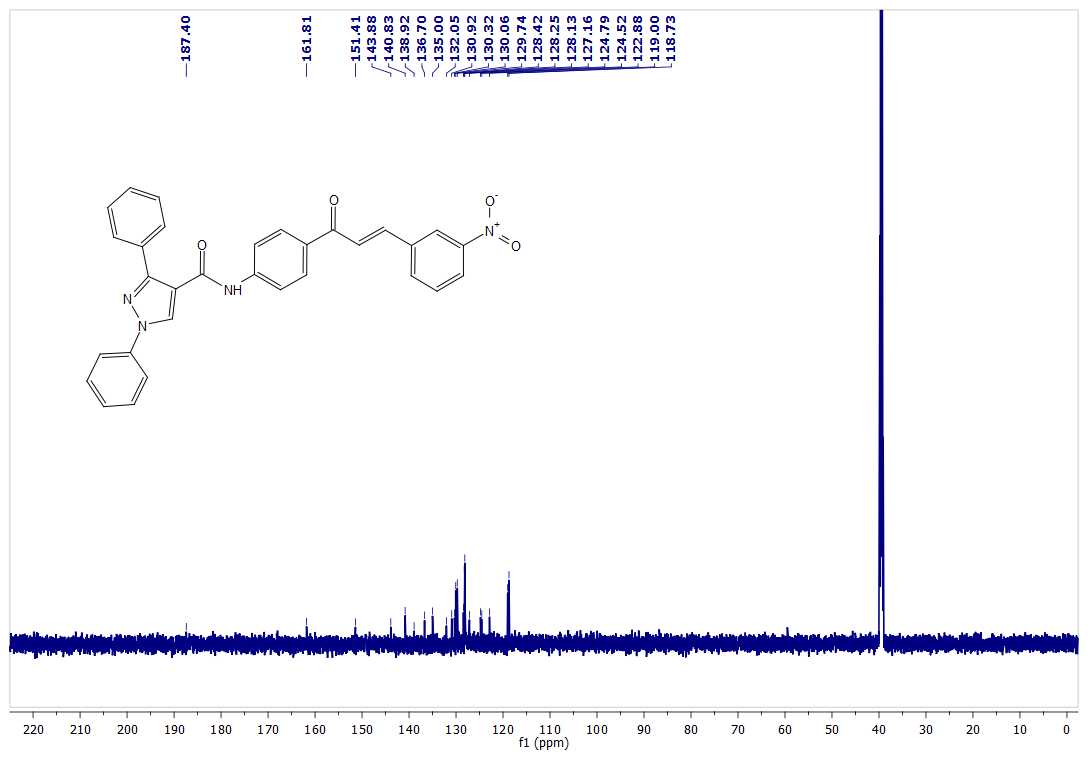
**

**^1^H NMR of 9l**

**
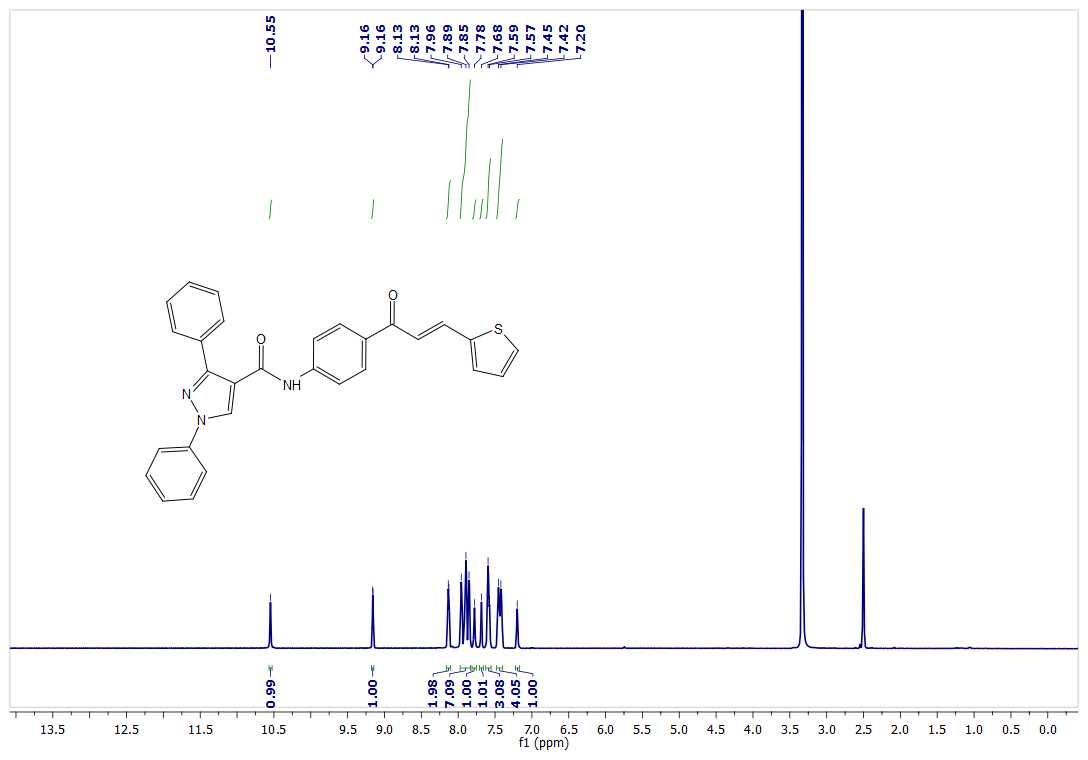
**

**^13^C NMR of 9l**

**
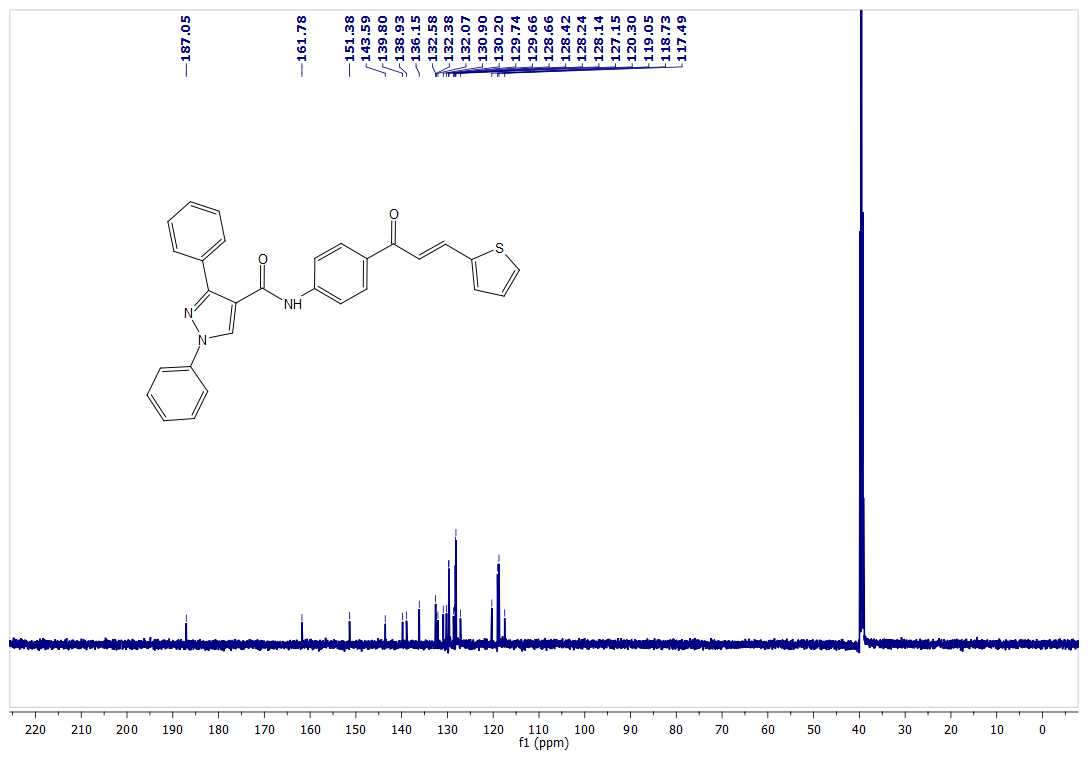
**

**^1^H NMR of 9m**

**
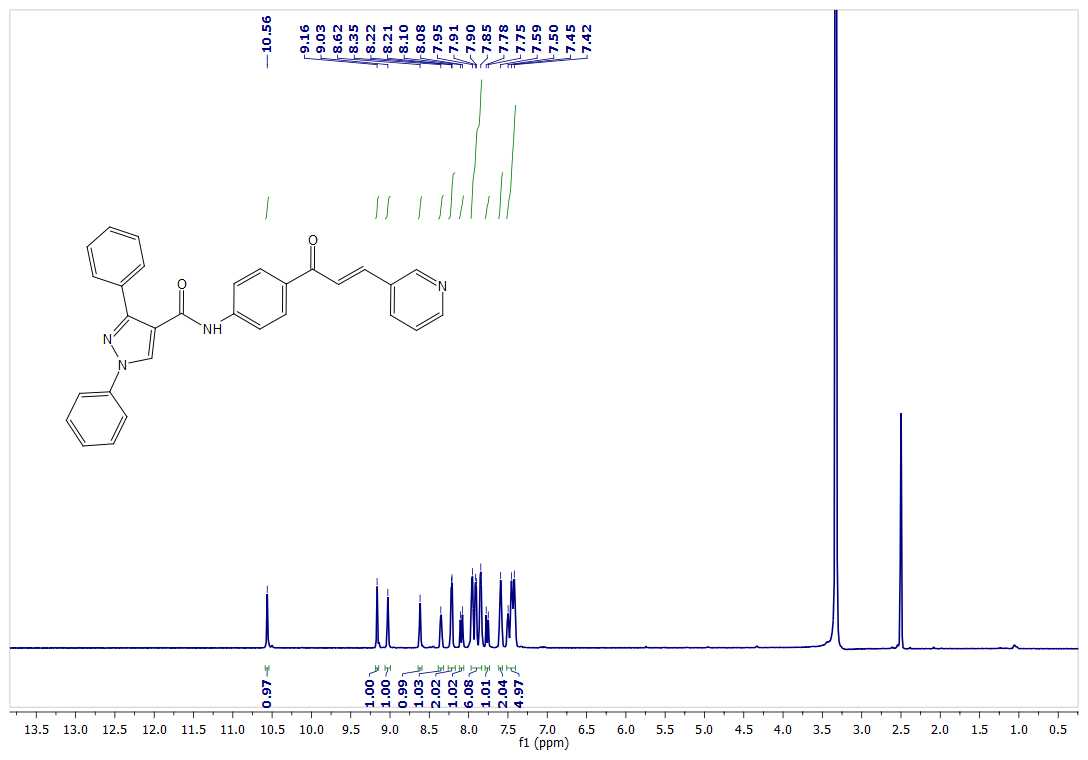
**

**^13^C NMR of 9m**

**
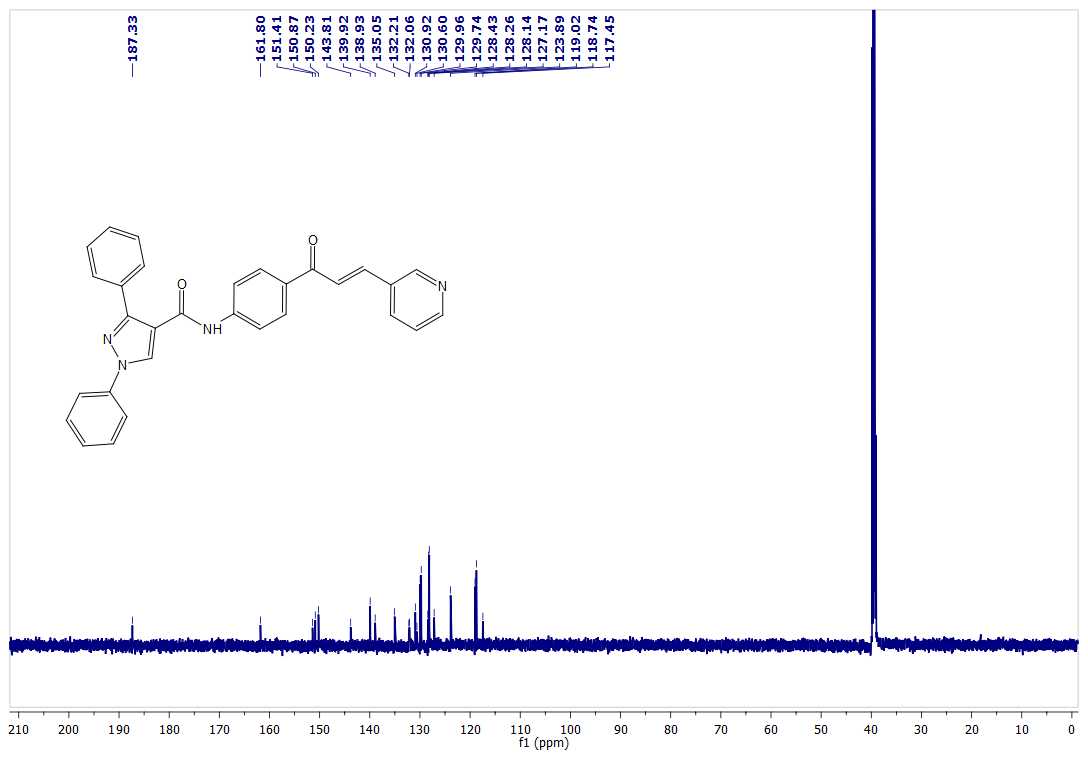
**

**^1^H NMR of 9n**

**
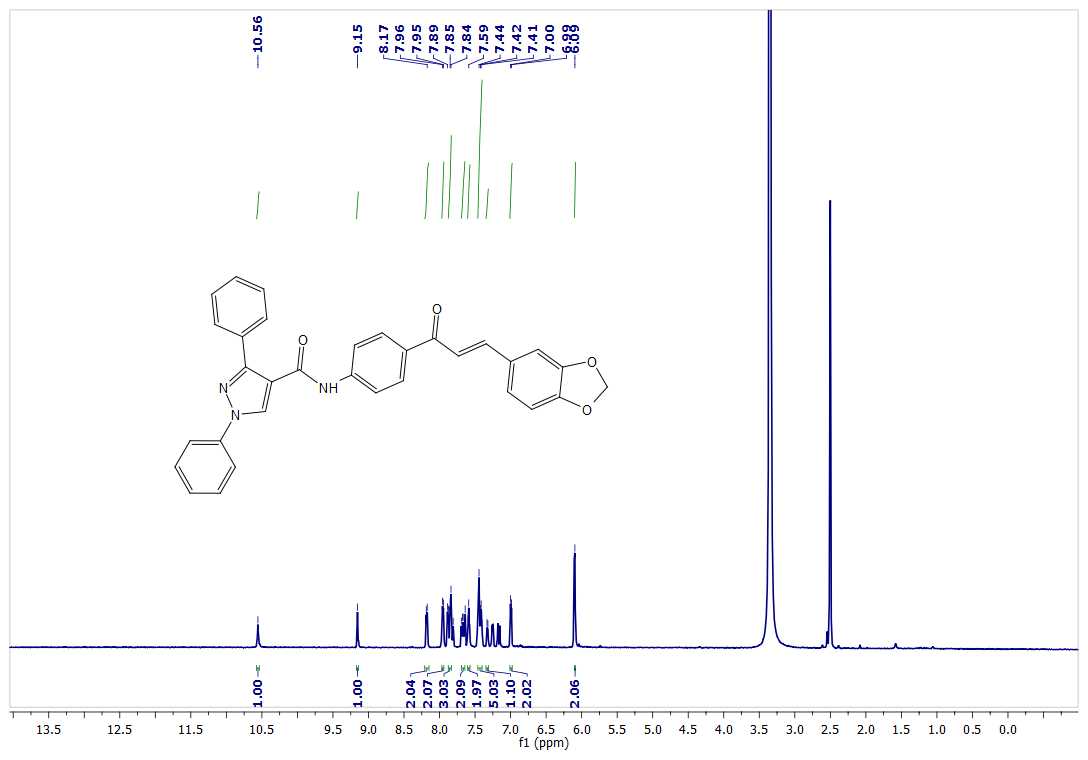
**

**^13^C NMR of 9n**

**
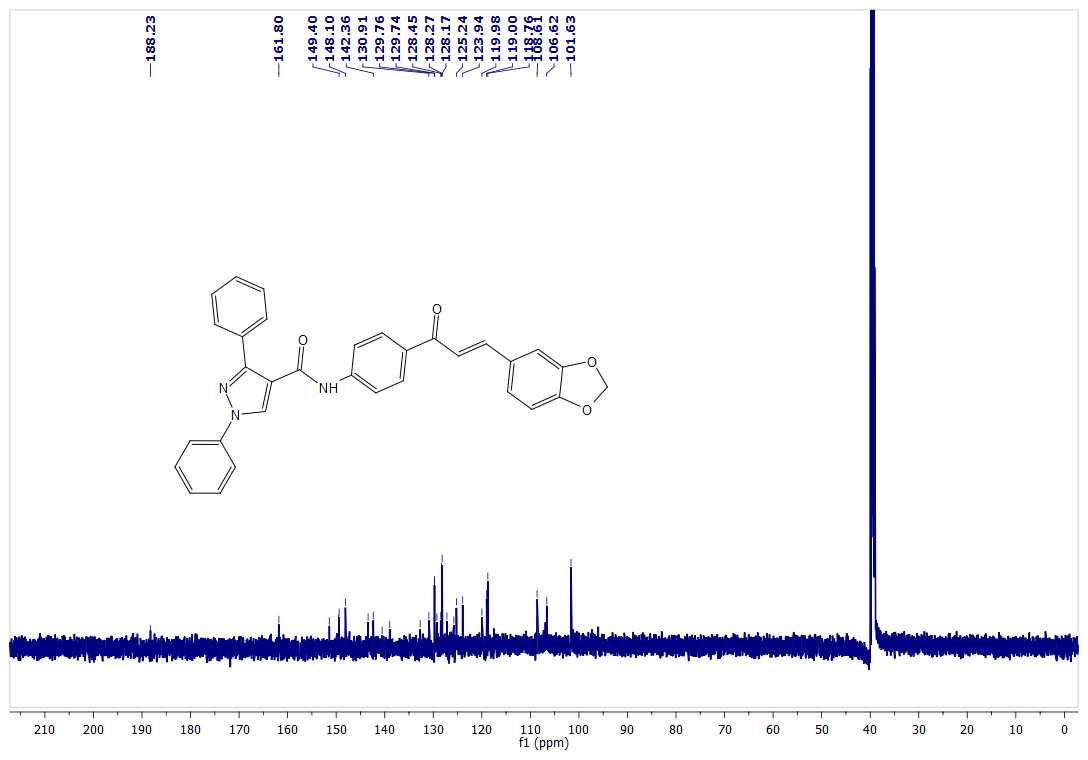
**

**HRMS spectra of 4**

**
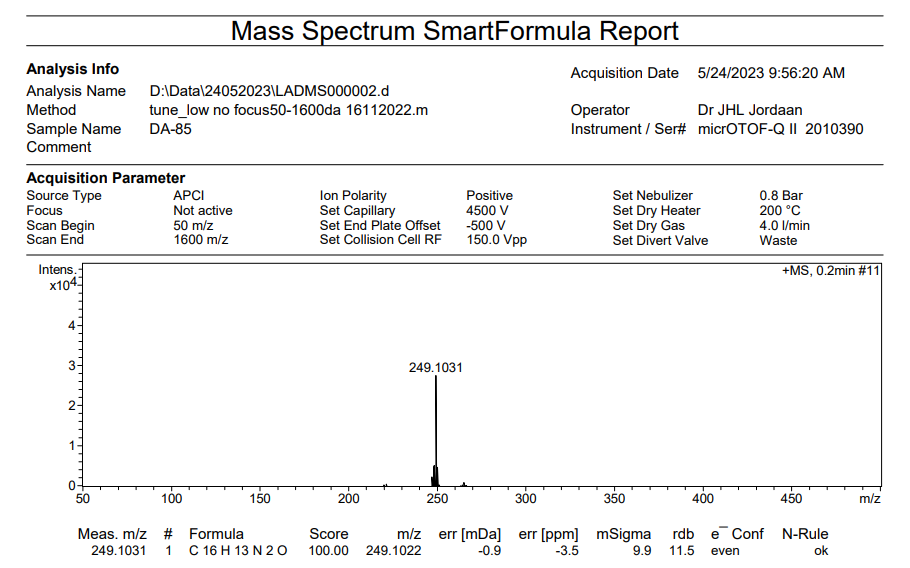
**

**HRMS spectra of 5**

**
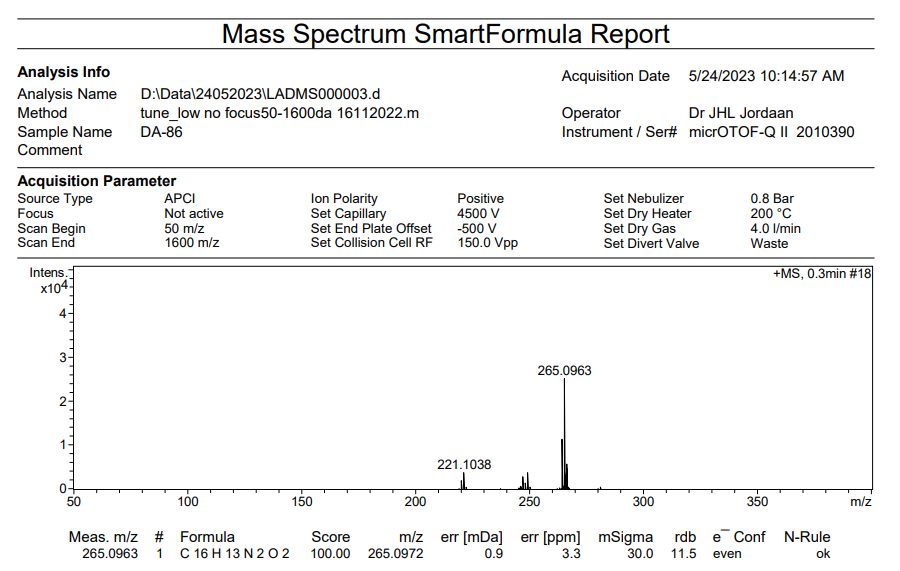
**

**HRMS spectra of 7**

**
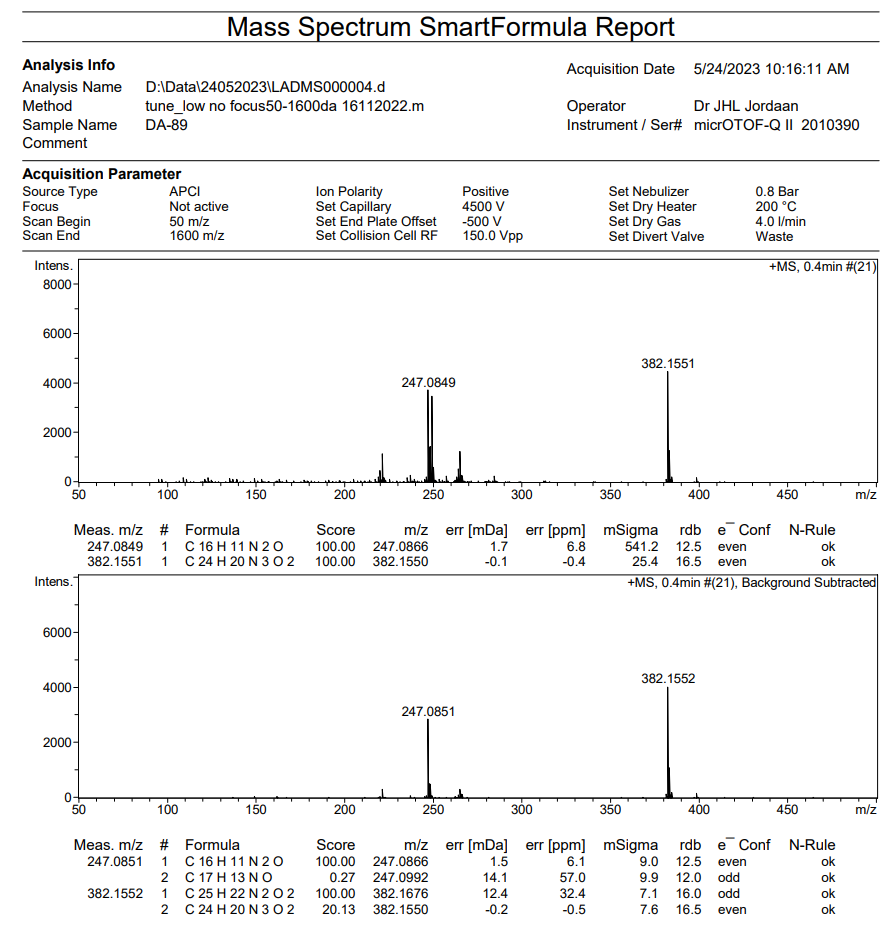
**

**HRMS spectra of 9a**

**HRMS spectra of 9b**

**HRMS spectra of 9c**

**HRMS spectra of 9d**

**
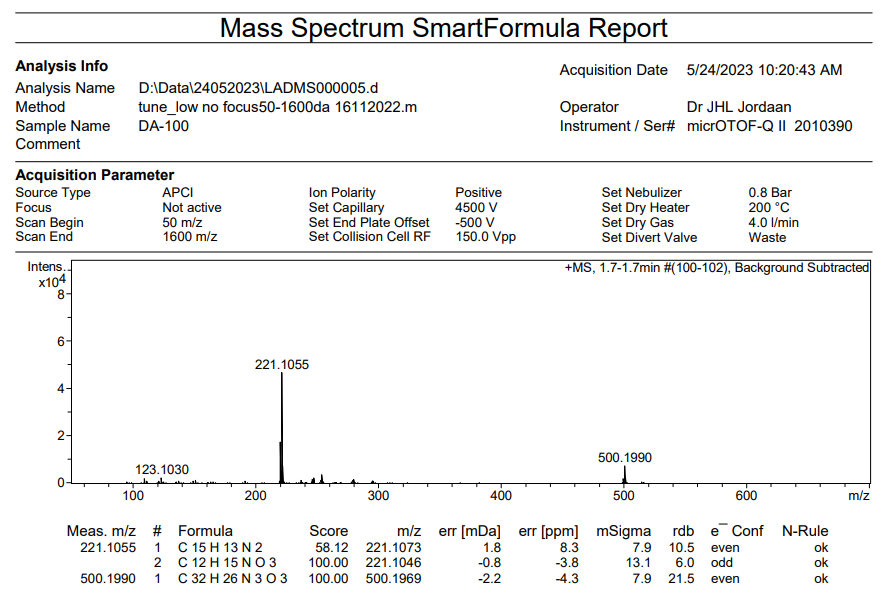
**

**HRMS spectra of 9e**

**HRMS spectra of 9f**

**
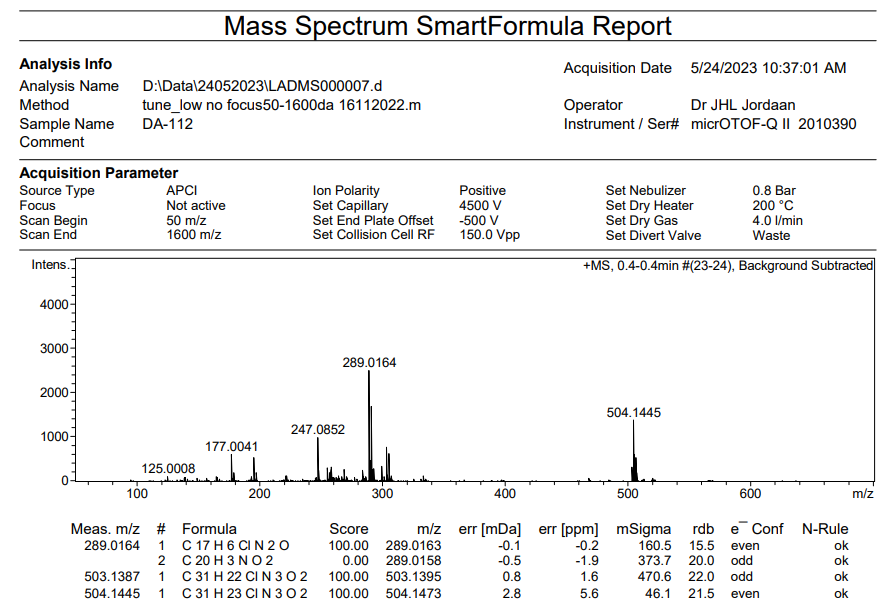
**

**HRMS spectra of 9g**

**
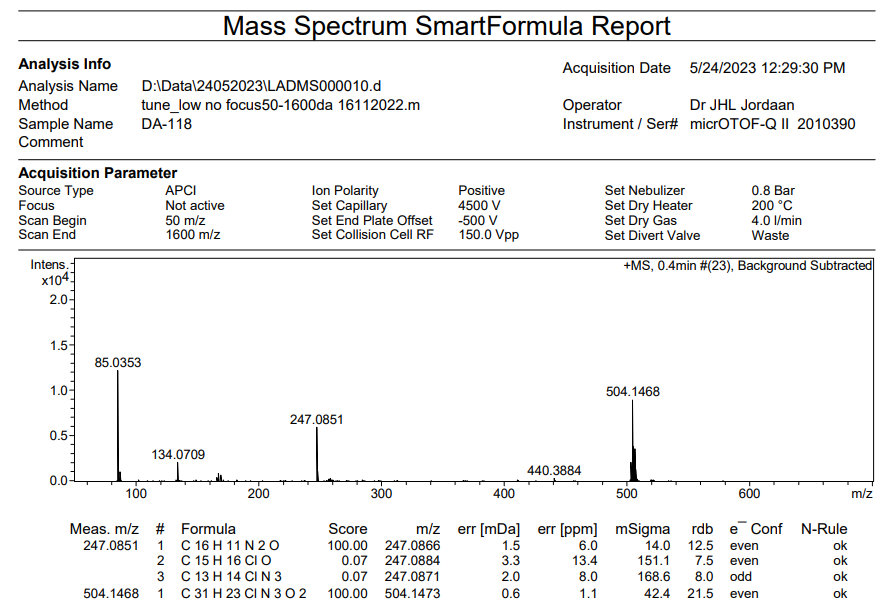
**

**HRMS spectra of 9h**

**
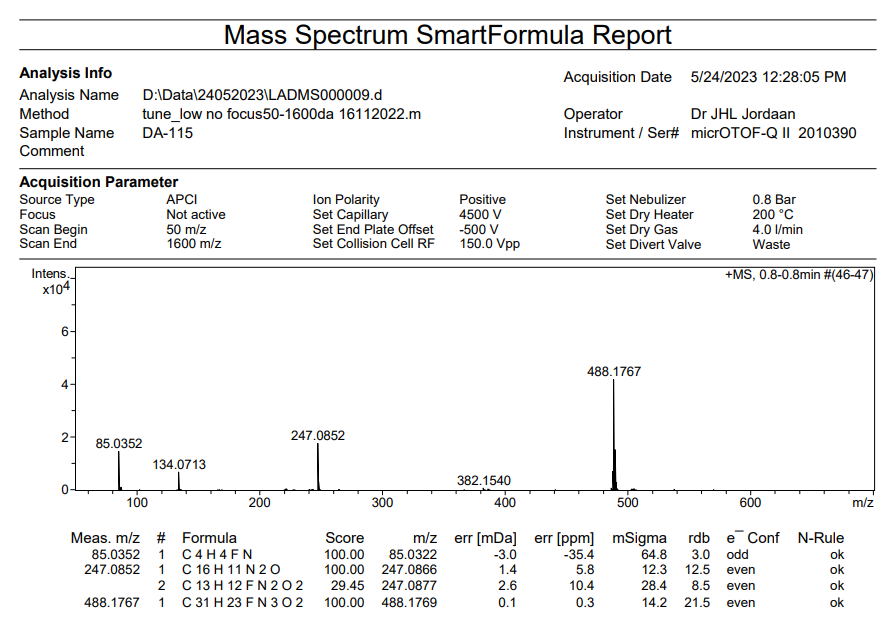
**

**HRMS spectra of 9i**

**
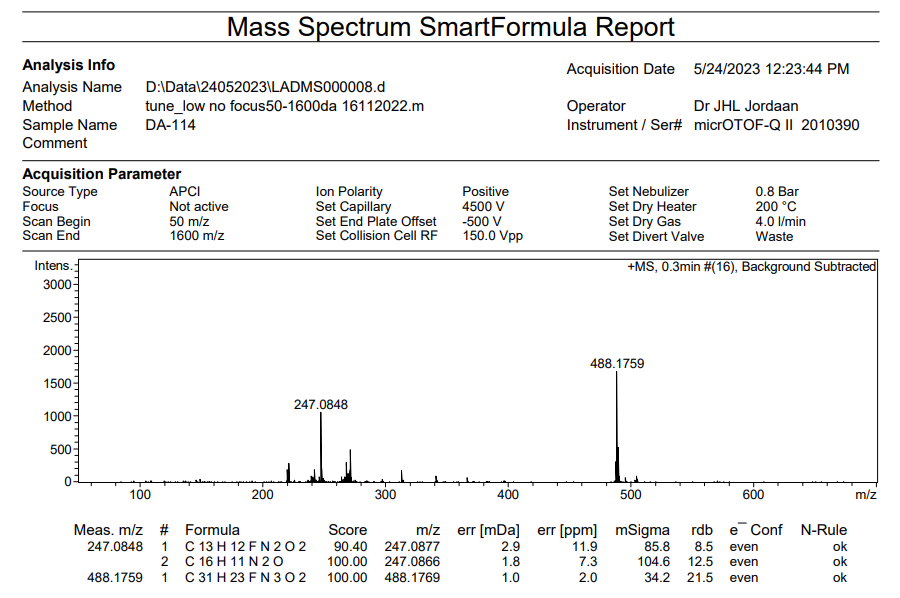
**

**HRMS spectra of 9j**

**
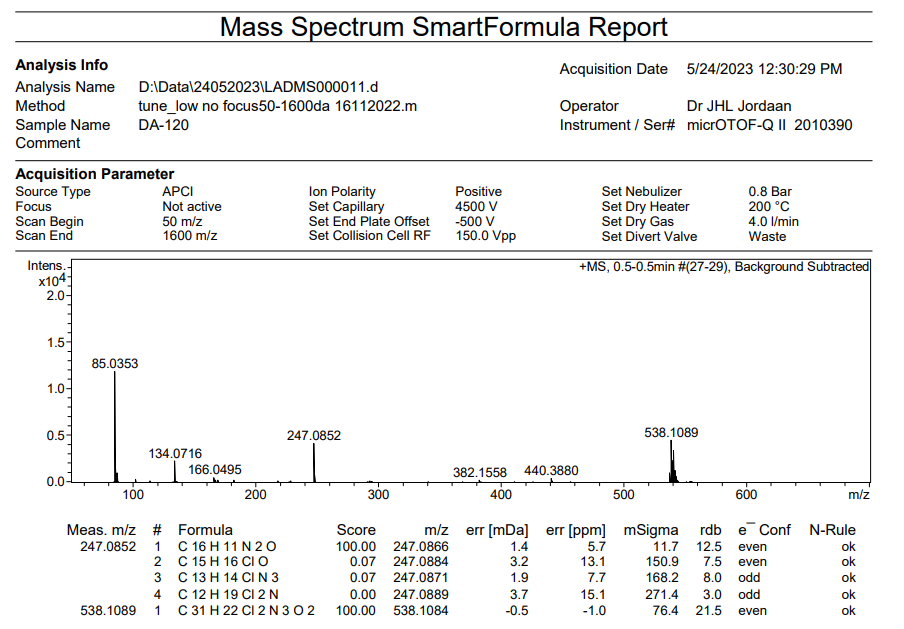
**

**HRMS spectra of 9k**

**
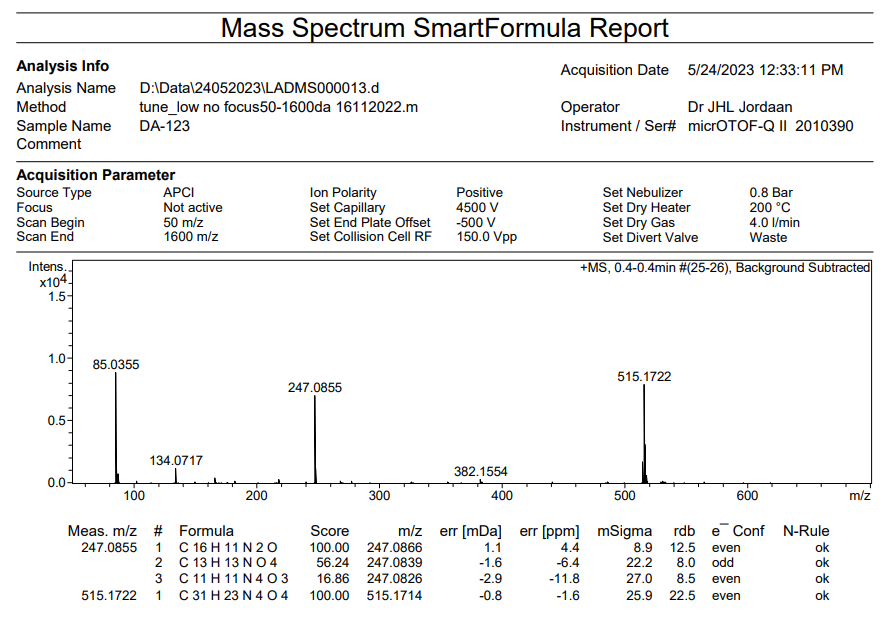
**

**HRMS spectra of 9l**

**
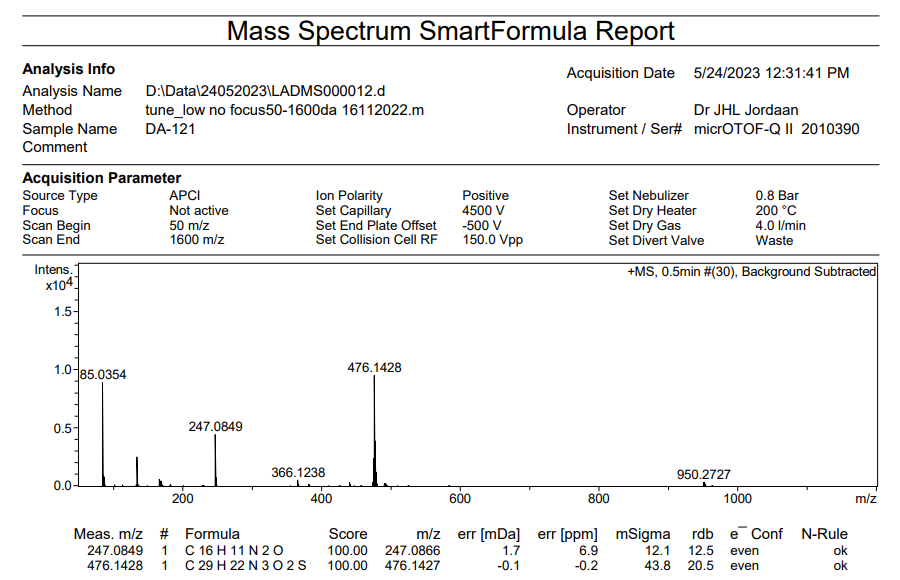
**

**HRMS spectra of 9m**

**
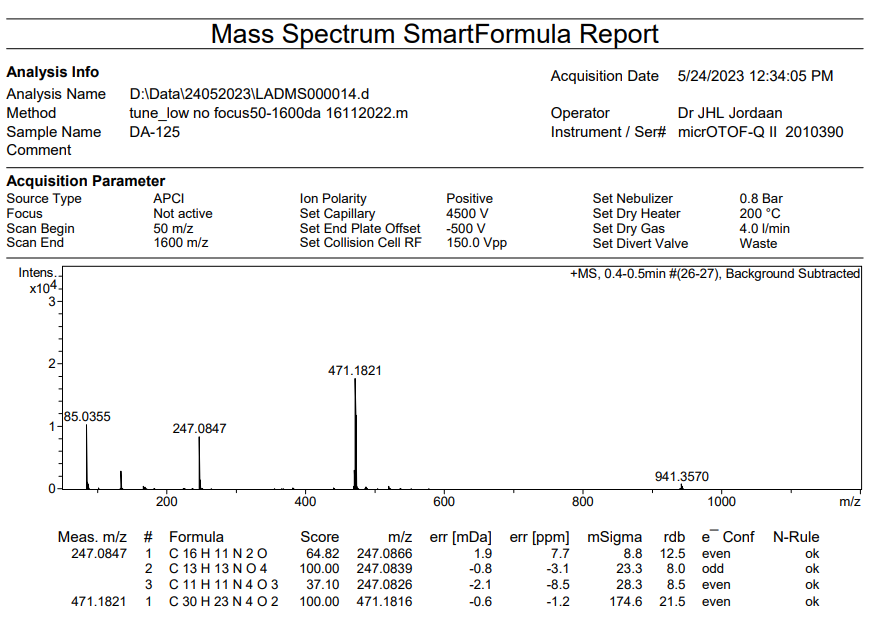
**

**HRMS spectra of 9n**
